# Bio‐Based Transparent Self‐Healable Polyimides Enabled by Isosorbide‐Derived Dianhydride Design and Aliphatic Chain‐Mobility Regulation

**DOI:** 10.1002/smll.75559

**Published:** 2026-09-03

**Authors:** Jisu Jang, Woojin Kang, Yeongseok Seo, Gyu Hyoung Bae, Jeong Yeon Yoo, Sang Goo Lee, Keun‐Hwan Oh, Hong Suk Kang

**Affiliations:** ^1^ Department of Polymer Science and Engineering and Program in Environmental and Polymer Engineering Inha University Incheon Republic of Korea; ^2^ Interface Material and Chemical Engineering Research Center Korea Research Institute of Chemical Technology Daejeon Republic of Korea; ^3^ Hydrogen Energy Research Center Korea Research Institute of Chemical Technology Daejeon Republic of Korea

## Abstract

Transparent self‐healable polyimides are attractive for flexible, wearable, and sustainable electronic devices, but achieving high transparency, rapid healing, and mechanical robustness within a single material remains challenging. Herein, we report a bio‐based aliphatic–aromatic polyimide platform that addresses this challenge through the cooperative molecular design of an isosorbide‐derived dianhydride (ISSDA), 4,4′‐oxydiphthalic anhydride, cystamine, and a bio‐based long‐chain aliphatic diamine (DDA). Unlike conventional aromatic polyimides, the nonplanar and nonconjugated ISSDA structure reduces charge‐transfer complex formation and prevents compact chain packing, enabling high optical transparency in a self‐healable polyimide network. The DDA segment further introduces controlled chain flexibility, lowers the effective healing barrier, and improves segmental mobility required for dynamic disulfide exchange. As a result, the optimized film achieved over 95% transmittance above 650 nm, thermal healing within 4 min at 150°C, and only a 7.8% loss in toughness after healing. DFT/TD‐DFT analyses confirmed that ISSDA suppresses and blueshifts the near‐UV electronic transitions relevant to visible transparency, while WAXD analysis revealed enlarged interchain *d*‐spacing and increased amorphous packing. This study provides a bio‐derived and structurally tunable route to transparent, tough, and rapidly self‐healable engineering polyimides.

## Graphical abstract

Transparency, toughness, and self‐healing rarely coexist within a single polyimide. This bio‐based aliphatic–aromatic film resolves that conflict. An isosorbide‐derived dianhydride and a fatty diamine cooperatively suppress charge‐transfer complexes, yielding a colorless film that heals rapidly under mild heat, retains its toughness, and restores conduction in embedded silver‐nanowire circuits. The design offers a general molecular strategy for sustainable engineering polyimides.

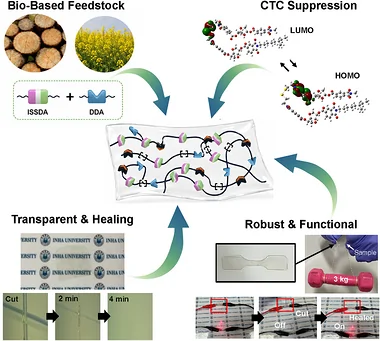

## 1 Introduction

Polyimides (PIs) are among the most representative high‐performance polymers because their rigid imide‐containing backbones and strong intermolecular interactions endow them with outstanding mechanical strength, thermal stability, and chemical resistance [1, 2]. Owing to these attributes, PI‐based materials have been widely used in flexible electronics, aerospace films, dielectric layers, and transparent substrates [1, 2, 3, 4, 5]. However, next‐generation flexible and wearable devices require more than intrinsically robust polymers. Under repeated operation, these systems are continuously exposed to bending, scratching, microcrack formation, and localized mechanical damage, which progressively deteriorate optical performance, interrupt electrical continuity, and ultimately compromise device reliability [1, 3, 6, 7, 8]. Accordingly, the development of self‐healable polyimides that can preserve the intrinsic thermal and chemical robustness of conventional PIs while autonomously recovering from damage has emerged as an important research direction [9, 10, 11, 12]. In particular, the development of self‐healable PIs from sustainable molecular building blocks is becoming increasingly important for extending their use in environmentally conscious flexible and wearable electronic systems.

For applications such as flexible displays, optical sensors, transparent protective coatings, and solar‐cell cover films, self‐healing functionality alone is not sufficient; high optical transparency must also be simultaneously achieved. A major challenge is that conventional aromatic PIs typically exhibit yellow to brown coloration because of charge‐transfer complex (CTC) formation between electron‐donating diamine moieties and electron‐accepting dianhydride units [1, 13]. Although the incorporation of bulky fluorinated groups or nonplanar structural motifs can reduce CTC formation and improve optical transparency, such modifications often weaken intermolecular cohesion and thus compromise mechanical stiffness or toughness [3, 13, 14, 15, 16, 17]. Likewise, the introduction of dynamic bonds and soft segments can enhance chain mobility and facilitate healing, but this approach frequently requires high healing temperatures and is often accompanied by reduced strength and modulus [6, 14, 18, 19, 20]. As a result, previously reported self‐healable PIs have generally suffered from intrinsic trade‐offs among transparency, healing capability, and mechanical robustness, often showing high glass‐transition temperatures, healing temperatures above 180°C, limited strain, and insufficient toughness [21, 22, 23, 24]. Therefore, a key unresolved challenge is to design a PI architecture in which CTC suppression, chain mobility, dynamic bond exchange, and mechanical integrity are controlled cooperatively rather than independently.

To address these conflicting requirements, we designed a bio‐based aliphatic–aromatic hybrid self‐healable PI system composed of isosorbide‐based dianhydride (ISSDA), 4,4′‐oxydiphthalic anhydride (ODPA), cystamine (Cys), and a bio‐based long‐chain aliphatic diamine (DDA, Priamine 1075). Notably, although isosorbide‐ and isomannide‐derived dianhydrides have been elegantly employed by Sawada and Ando to achieve transparent, low‐dielectric semi‐alicyclic polyimides [2, 25, 26], this work represents, to our knowledge, the first introduction of such an isohexide‐derived dianhydride (ISSDA) into a self‐healable polyimide system, establishing ISSDA not merely as a bio‐based monomer but as a functional structural unit for suppressing CTC formation, disrupting dense chain packing, and enhancing optical transparency. The molecular design is based on four complementary roles. First, ISSDA provides a nonplanar, V‐shaped three‐dimensional structure derived from an isosorbide‐based bicyclic acetal/ether motif, which enlarges free volume, suppresses dense chain packing, and weakens CTC formation, thereby promoting high optical transparency [25, 26, 27, 28]. This structural feature differentiates ISSDA from conventional aromatic dianhydrides and provides a sustainable alternative to transparency‐enhancing strategies that rely heavily on fluorinated or highly bulky monomers. Second, ODPA serves as a relatively flexible aromatic dianhydride that helps balance rigidity and ductility within the copolyimide framework. Third, Cys introduces disulfide bonds that enable dynamic covalent exchange, providing a chemical pathway for self‐healing [6]. Fourth, DDA contains long aliphatic chains that enhance segmental mobility, lower the effective healing‐temperature window, and facilitate rapid interfacial rearrangement during the early stage of healing [2]. In this system, DDA is not simply used as a soft segment; rather, it acts as a molecular mobility regulator that adjusts the glass‐transition behavior and assists dynamic disulfide exchange by increasing local chain motion. Because both ISSDA and DDA originate from bio‐based feedstocks, this material platform also offers a sustainable route toward high‐performance functional polyimides [2, 29].

In this study, we show that the proposed molecular design goes beyond the simple preparation of a transparent self‐healable PI. Rather, it integrates several previously conflicting features within a single polymer system: (i) high transparency through CTC suppression, (ii) rapid healing assisted by free‐volume‐rich chain packing and enhanced chain mobility, (iii) efficient damage recovery enabled by the synergy between disulfide exchange and physical chain rearrangement, and (iv) retention of mechanical toughness that is often sacrificed in transparent self‐healing PIs. The cooperative roles of ISSDA and DDA were further verified by optical, thermal, mechanical, DFT/TD‐DFT, and WAXD analyses, which link the macroscopic transparency and healing performance to monomer‐level electronic‐structure modulation and supramolecular packing control. Furthermore, the present system demonstrates proof‐of‐concept electrical function recovery when combined with a silver nanowire conductive network, suggesting its potential as a functional substrate for flexible electronics. The optimized composition exhibited approximately 88% transmittance at 550 nm and over 95% transmittance above 650 nm, along with rapid healing at 150°C within 4 min and only a 7.8% reduction in toughness after healing. These results demonstrate that the long‐standing trade‐off among transparency, healing performance, and mechanical durability in self‐healable polyimides can be effectively alleviated through precise monomer‐level design of a bio‐based aliphatic–aromatic hybrid architecture.

## 2 Results and Discussion

### 2.1 Molecular Design Strategy for Transparent and Self‐Healable Polyimides

Figure 1a illustrates the molecular structures of ISSDA, ODPA, Cys, and DDA, together with the overall design concept of the bio‐based aliphatic–aromatic self‐healable polyimides developed in this study. The central strategy of this work was not simply to combine a dynamic diamine with a flexible segment, but to construct a monomer platform in which a bio‐based nonplanar dianhydride and a bio‐based long‐chain aliphatic diamine cooperatively regulate optical transparency, chain mobility, and healing behavior within a single polyimide architecture. In this respect, the introduction of both ISSDA and DDA represents a key point of distinction from previously reported transparent self‐healable polyimides, which have largely relied on a limited set of conventional dianhydrides such as BPADA or fluorinated analogues to tune transparency and flexibility [1, 3, 14].

**FIGURE 1:**
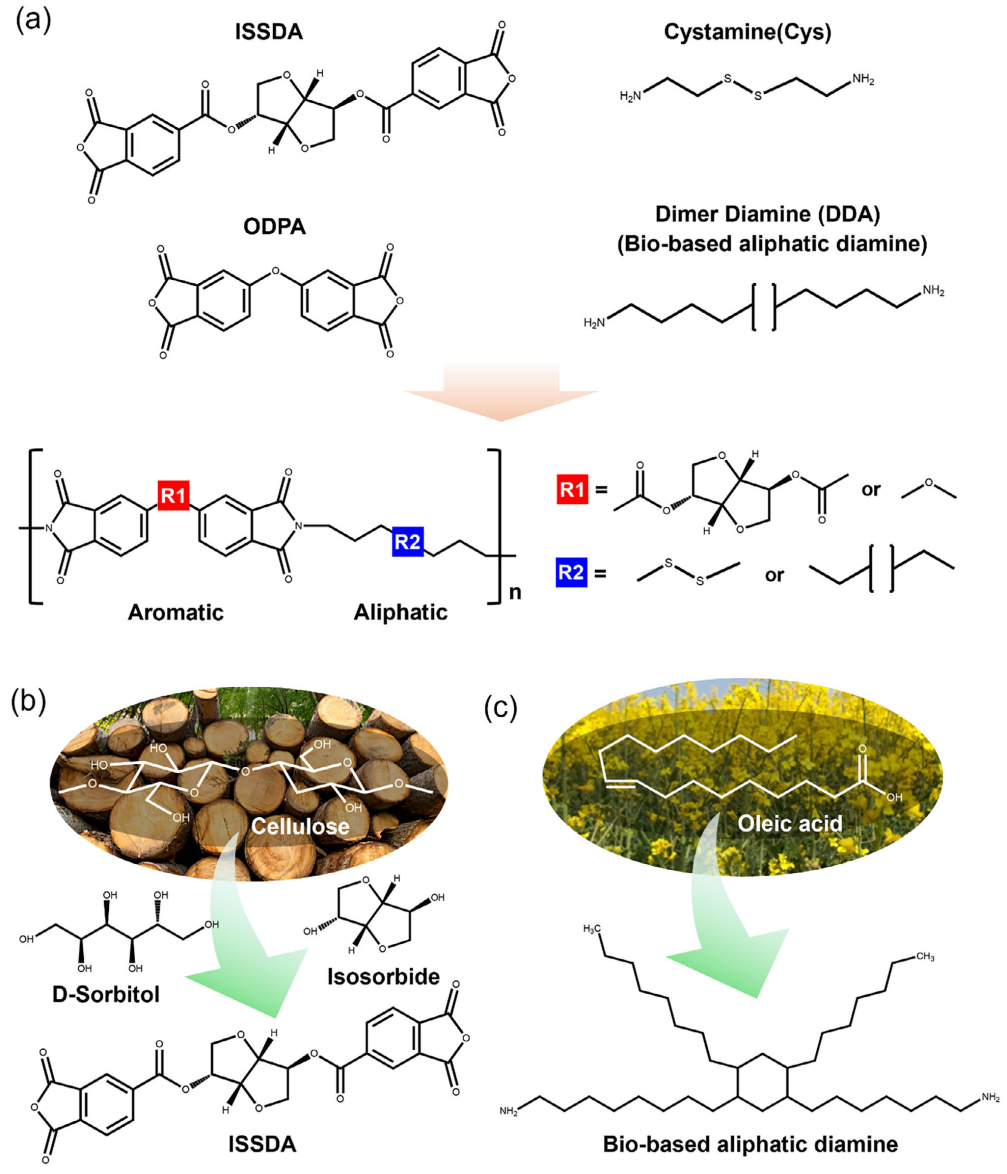
Molecular structures and sustainable design concept of bio‐based aliphatic–aromatic self‐healable polyimides. (a) Chemical structures of the monomers employed in this study: 4,4′‐oxydiphthalic anhydride (ODPA), isosorbide‐based dianhydride (ISSDA), cystamine (Cys), and bio‐based aliphatic diamine (DDA). Schematic representation of the copolyimide architecture, in which the dianhydride segment (R1) and diamine segment (R2) are systematically varied to integrate optical transparency, chain mobility, and dynamic healing capability. (b) Renewable feedstock origin of ISSDA, derived from cellulose‐based isosorbide via D‐sorbitol. (c) Renewable feedstock origin of DDA, derived from oleic acid.

Among the monomers employed, ISSDA was selected as a particularly important structural component because it combines an isosorbide‐derived nonplanar bicyclic framework with trimellitic anhydride units. Unlike conventional planar aromatic dianhydrides, ISSDA possesses a bent and sterically bulky three‐dimensional geometry that can suppress dense chain packing and reduce charge‐transfer complex formation while still maintaining sufficient backbone rigidity [25, 26]. This structural feature makes ISSDA especially attractive for the design of transparent polyimides, because the reduction of interchain electronic interactions is expected to improve optical clarity without completely sacrificing the inherent robustness of the imide backbone. As shown in Figure 1b, ISSDA originates from isosorbide, which is derived from D‐sorbitol via cellulose‐based biomass, providing a sustainability advantage in addition to its structural role [25, 29].

ODPA was introduced as a complementary aromatic dianhydride to balance the excessive rigidity that could arise from the use of a highly contorted dianhydride alone. Owing to its ether linkage, ODPA can provide a more flexible aromatic segment and thereby help maintain film‐forming ability and ductility within the copolyimide framework. Accordingly, ODPA served not only as a compositional counterpart to ISSDA but also as a useful structural reference for clarifying how the incorporation of ISSDA influences transparency, free volume, and healing‐related chain rearrangement in the resulting polyimides.

On the diamine side, Cys and DDA were designed to play distinct but cooperative roles. Cys contains disulfide bonds that can undergo dynamic covalent exchange, thereby providing the essential chemical pathway for self‐healing [6]. In contrast, DDA functions primarily as a soft aliphatic diamine that promotes segmental mobility and interfacial chain rearrangement through its long hydrocarbon chain. Compared with short or relatively rigid diamines, the incorporation of DDA was expected to lower the glass‐transition‐related healing window and facilitate faster recovery under milder thermal conditions [2]. Moreover, the use of DDA is important not only from the viewpoint of flexibility but also from the viewpoint of molecular design novelty, because this long‐chain fatty diamine, derived from bio‐based feedstocks (*e.g*., oleic‐acid‐based resources), introduces a distinct aliphatic domain into the polyimide backbone [2]. We note that the optical transparency and thermal/mechanical properties of the parent ISSDA‐DDA homopolyimide have been reported in detail [2]; the present work builds on this established platform by further incorporating ODPA to modulate compositional balance and processability, and Cys to introduce dynamic disulfide‐based self‐healing functionality, neither of which was present in the previously reported system. This feature differentiates the present system from more conventional transparent self‐healing PI designs that mainly depend on aromatic or fluorinated monomer platforms [1, 14].

Figure 1b,c further highlights that the present molecular design is closely connected to renewable feedstock platforms. The dianhydride platform is built around an isosorbide‐derived monomer, whereas the long‐chain diamine segment originates from a bio‐based fatty diamine. In support of this design concept, Figure S1 presents the synthetic route for ISSDA. Figure S2 shows the ^1^H NMR spectrum of the synthesized ISSDA, confirming the successful formation of the target dianhydride structure. Figure S3 summarizes the physicochemical characteristics of DDA (Priamine 1075), including amine value, molecular weight, dimer content, and dynamic viscosity. Figure S4 provides the ^1^H NMR spectrum of the synthesized cystamine, verifying its chemical identity prior to polymerization. Figure S5 shows the FT–IR spectra of polyimide films at varying ISSDA:ODPA compositions with a fixed Cys:DDA ratio of 5:5. The characteristic imide carbonyl stretching bands at approximately 1780 and 1720 cm^−^^1^ and the imide ring deformation band near 720 cm^−^^1^ are clearly present across all compositions, confirming successful thermal imidization regardless of dianhydride ratio. These demonstrate that the present system is not merely based on conceptual monomer selection, but is supported by the practical synthesis and integration of key bio‐based and dynamic building blocks into the polyimide backbone. Quantitatively, based on the known bio‐ or fossil‐derived origin of each monomer's carbon atoms, the optimized composition is calculated to have a theoretical bio‐based carbon content of approximately 52.9% (Section S5).

From a structure–property perspective, each monomer was assigned a distinct function within the polymer architecture. ISSDA was responsible mainly for transparency enhancement and free‐volume generation through its nonplanar three‐dimensional geometry. ODPA contributed to compositional balance and ductility through its flexible aromatic ether linkage. Cys provided the dynamic disulfide exchange sites required for chemical healing. DDA contributed to chain mobility, low‐temperature healing capability, and improved extensibility through its long aliphatic segment. Therefore, the present polymer system should be understood not as a random copolyimide composition, but as a deliberately integrated molecular platform in which transparency, healing, and mechanical compliance are distributed across different monomer units and then synergistically combined.

The most important difference between the present design and earlier transparent self‐healing PI systems lies in the fact that transparency and healing were not tuned independently. Instead, both properties were co‐engineered through the simultaneous introduction of a bio‐based contorted dianhydride and a bio‐based long‐chain diamine. ISSDA weakens CTC formation and expands intermolecular spacing, while DDA increases segmental mobility and facilitates rapid interfacial recovery; when combined with the disulfide exchange chemistry of Cys, these effects create a favorable environment for transparent and fast‐healing polyimides with improved toughness retention. In other words, the present molecular design is distinguished by the cooperative use of ISSDA and DDA as two uncommon yet highly effective monomeric building blocks, enabling the mitigation of the typical trade‐off among optical transparency, self‐healing efficiency, and mechanical robustness in transparent polyimide systems.

### 2.2 Optical Transparency, Rapid Self‐Healing, and Proposed Healing Mechanism

Figure 2 comprehensively summarizes the optical transparency, self‐healing performance, and proposed molecular healing mechanism of the bio‐based aliphatic–aromatic polyimides developed in this study. Figure 2a presents the UV–vis transmittance spectra of polyimide films with varying ISSDA:ODPA molar ratios at a fixed Cys:DDA ratio of 5:5. A clear and systematic trend is evident: as the ISSDA content increased, the visible‐light transmittance of the films improved progressively across the entire measured wavelength range. The film with the highest ISSDA content (ISSDA:ODPA = 6:4) exhibited the greatest transmittance, reaching approximately 88% at 550 nm and exceeding 95% in the longer visible wavelength region above 650 nm. In contrast, the ODPA‐only composition (ISSDA:ODPA = 0:10) showed markedly lower transparency, with a transmittance of approximately 65% at 550 nm, accompanied by a pronounced absorption onset shifted toward longer wavelengths relative to the ISSDA‐rich compositions. The intermediate compositions (ISSDA:ODPA = 4:6 and 2:8) displayed transmittance values of approximately 83% and 69% at 550 nm, respectively, confirming a monotonic composition–transparency relationship. These results indicate that the improvement in optical transparency cannot be attributed simply to the presence of aliphatic diamine segments, but is strongly associated with the incorporation of ISSDA, whose nonplanar and sterically bulky structure can effectively suppress dense interchain packing and charge‐transfer complex (CTC) formation [25, 26].

**FIGURE 2:**
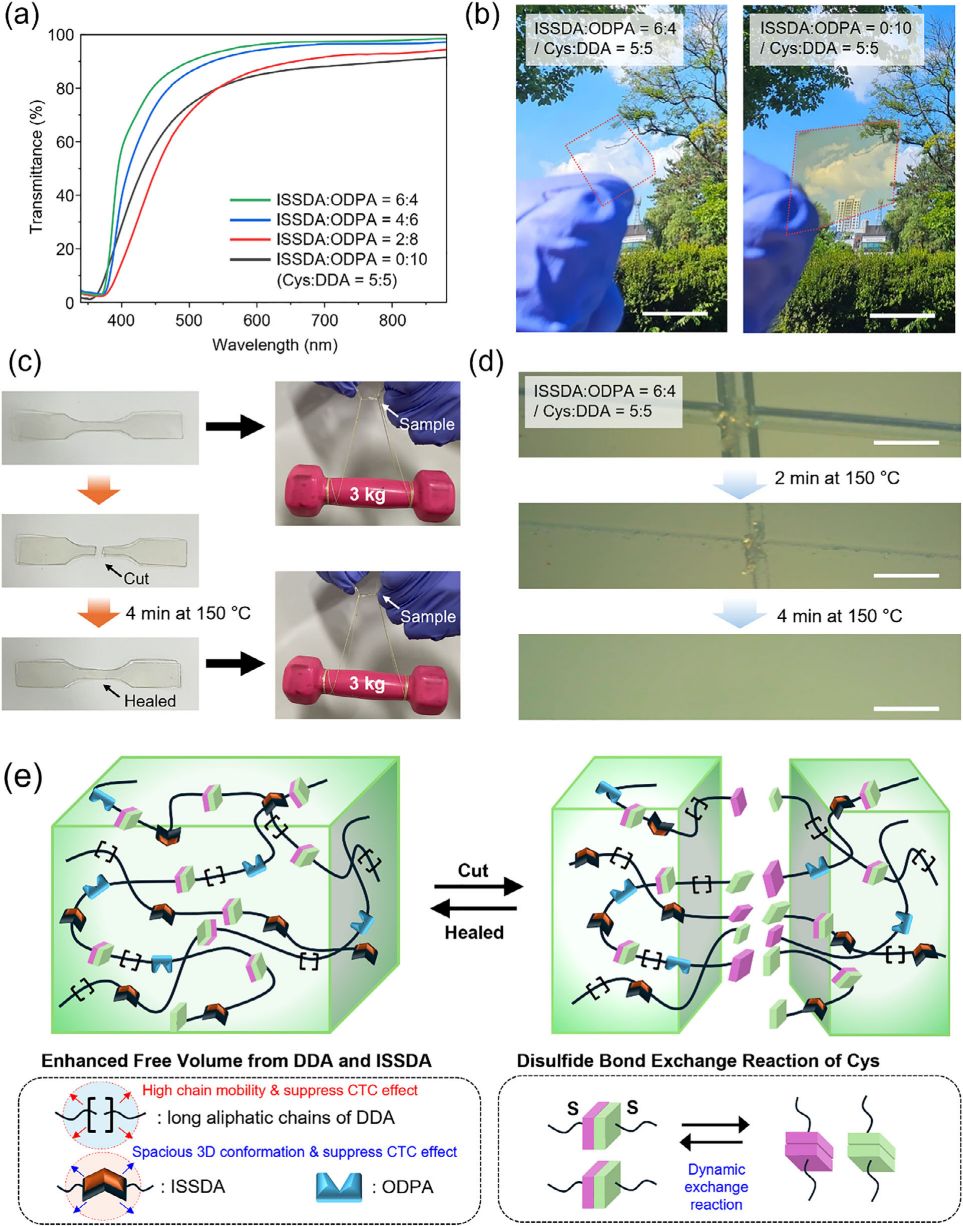
Optical transparency, self‐healing performance, and proposed healing mechanism of the bio‐based aliphatic–aromatic polyimides. (a) UV–vis transmittance spectra of polyimide films with different ISSDA:ODPA compositions at a fixed Cys:DDA ratio of 5:5. (b) Photographs comparing the optical transparency of polyimide films with ISSDA:ODPA = 6:4 and 0:10 at the same Cys:DDA ratio of 5:5. (c) Photographs showing the cut‐and‐heal process of the dog‐bone‐shaped polyimide specimen and the load‐bearing capability of the healed film under a 3 kg weight. (d) Optical microscopy images of the damaged region before and after healing for the polyimide film with ISSDA:ODPA = 6:4 and Cys:DDA = 5:5. (scale bars: 500 µm) (e) Schematic illustration of the molecular origin of the enhanced self‐healing behavior, highlighting the roles of DDA‐induced high chain mobility, ISSDA‐induced spacious 3D conformation and suppressed CTC effect, and disulfide exchange reactions from Cys.

The same trend is also clearly visible in the photographic comparison shown in Figure 2b. Under the same diamine composition of Cys:DDA = 5:5, the ISSDA‐containing film (ISSDA:ODPA = 6:4) was sufficiently transparent to allow the background image to be clearly observed, whereas the ODPA‐based counterpart appeared relatively hazy and more yellowish. This difference is more clearly confirmed in the enlarged photographs shown in Figure S6, which were taken to more distinctly visualize the transparency difference between the two films. Thus, the role of ISSDA is not limited to a marginal spectral improvement, but extends to a visually distinguishable enhancement in optical clarity. This observation further supports the view that ISSDA acts as a key structural motif for transparent self‐healable PI design rather than as a minor comonomer modifier.

The self‐healing capability of the optimized film is directly demonstrated in Figure 2c. After the dog‐bone‐shaped specimen was cut, the separated parts were rejoined by thermal treatment, and the healed sample recovered its continuous shape. More importantly, the healed film was able to support a 3 kg load, indicating that the healing process restored not only superficial contact but also substantial mechanical integrity across the damaged interface. In self‐healing polymer systems, visual crack disappearance alone does not necessarily guarantee effective stress transfer after healing [6, 14]. Therefore, the load‐bearing result in Figure 2c provides direct macroscopic evidence that the present polymer system can recover a mechanically functional interface after severe damage.

At the microscopic level, Figure 2d provides optical microscopy images documenting the time‐resolved crack closure process for the same optimized composition. The initially sharp and clearly defined damaged region began to close upon heating at 150°C, and the crack became nearly indistinguishable after healing at 150°C for 4 min. These rapid closure kinetics are noteworthy in comparison with many previously reported self‐healing PI systems, which typically require higher healing temperatures (often above 200°C) and/or substantially longer healing durations [1, 6, 18, 30, 31]. The rapid closure behavior is particularly important because it suggests that the healing process is not limited by extremely slow chain rearrangement, but instead proceeds efficiently once sufficient thermal activation is provided.

The molecular origin of this behavior is schematically illustrated in Figure 2e. DDA, with its long aliphatic chain, enhances segmental mobility and facilitates interfacial rearrangement during healing [2]. At the same time, ISSDA introduces a spacious three‐dimensional nonplanar structure that suppresses CTC formation while increasing free volume and reducing excessive chain packing. Cys provides dynamic disulfide bonds that can undergo exchange reactions at the damaged interface, thereby enabling chemical healing. In this regard, the healing behavior of the present system should not be understood as the result of a single factor, but rather as a synergistic process in which dynamic covalent exchange operates within a polymer matrix that has been structurally engineered to possess sufficient mobility and free‐volume‐assisted interfacial accessibility.

Taken together, Figure 2 demonstrates that the present molecular design successfully integrates high transparency and rapid self‐healing within one polyimide platform. The enhanced transparency originates primarily from ISSDA‐induced suppression of dense interchain electronic interactions, whereas the fast healing behavior is enabled by the combined effects of DDA‐induced chain mobility, ISSDA‐derived free‐volume‐rich packing, and Cys‐mediated disulfide exchange. The key design insight of this system is therefore not the simple introduction of a dynamic functional group, but rather the cooperative structural optimization of optical properties, segmental dynamics, and dynamic bond chemistry through monomer‐level molecular engineering within a bio‐based aliphatic–aromatic polyimide framework. This interpretation is further supported by the DFT/TD‐DFT and WAXD analyses discussed below: TD‐DFT calculations show that ISSDA suppresses and blueshifts the near‐UV electronic transitions relevant to visible transparency, while WAXD analysis independently shows that ISSDA enlarges the average interchain spacing, together providing an electronic and packing‐level basis for the experimentally observed transparency enhancement.

### 2.3 Origin of Optical Transparency: CTC Suppression, Reduced Transition Intensity, and Enlarged Intermolecular Spacing

Figure 3a shows the DFT‐optimized single‐chain model of the ODPA‐based segment, composed of ODPA, Cys, ODPA, and DDA units connected in sequence. The TD‐DFT‐calculated absorption spectrum of the ODPA‐based model, obtained at CAM‐B3LYP/6‐311G(d,p)//B3LYP/6‐311+G(2d,p) over the fifty lowest singlet excited states, is shown in Figure 3b. The spectrum shows a strong, dominant band near 209 nm (f = 0.856), together with a weaker secondary band near 287 nm (f = 0.149) that lies closer to the near‐UV/visible boundary relevant to film transparency.

**FIGURE 3:**
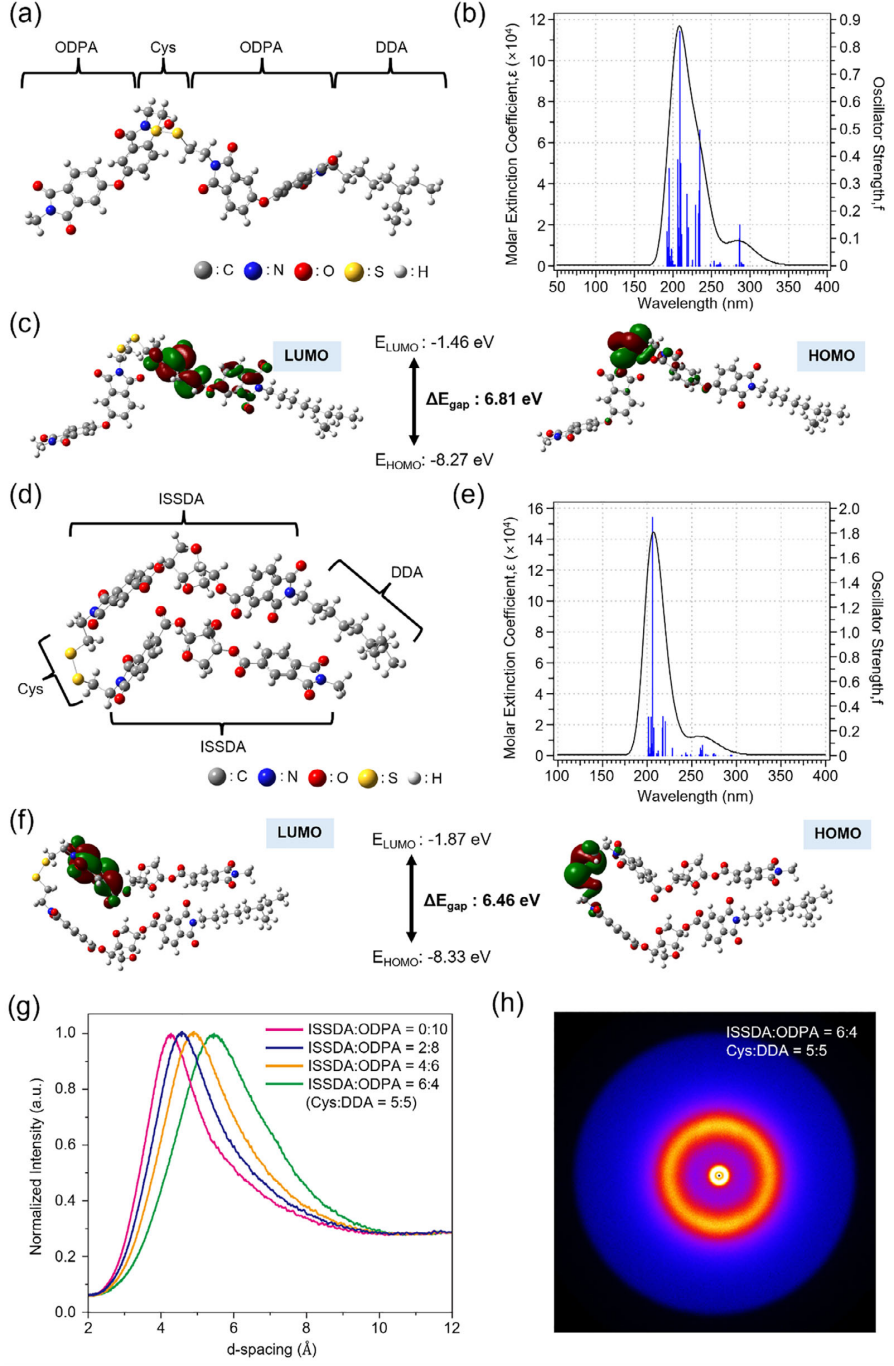
DFT/TD‐DFT and WAXD analyses of the molecular origin of enhanced optical transparency in the bio‐based aliphatic–aromatic polyimides. (a) DFT‐optimized single‐chain molecular model of the ODPA‐based polyimide segment composed of ODPA, Cys, ODPA, and DDA units. (b) TD‐DFT‐calculated UV–vis absorption profile of the ODPA‐based model (CAM‐B3LYP/6‐311G(d,p)//B3LYP/6‐311+G(2d,p), 50 lowest singlet states). (c) Frontier molecular orbital distributions and energy levels of the ODPA‐based single‐chain model, showing a HOMO–LUMO gap of 6.81 eV. (d) DFT‐optimized single‐chain molecular model of the ISSDA‐based polyimide segment composed of Cys, ISSDA, ISSDA, and DDA units. (e) TD‐DFT‐calculated UV–vis absorption profile of the ISSDA‐based model. (f) Frontier molecular orbital distributions and energy levels of the ISSDA‐based single‐chain model, showing a HOMO–LUMO gap of 6.46 eV. (g) WAXD profiles of polyimide films with different ISSDA:ODPA compositions at a fixed Cys:DDA ratio of 5:5. (h) Two‐dimensional WAXD pattern of the optimized polyimide film (ISSDA:ODPA = 6:4, Cys:DDA = 5:5), showing an isotropic amorphous halo.

Figure 3c visualizes the frontier molecular orbitals of the ODPA‐based single‐chain model. The calculated HOMO and LUMO energy levels are −8.27 and −1.46 eV, respectively, giving a HOMO–LUMO gap of 6.81 eV. Both orbitals are co‐localized near the Cys disulfide linkage and its adjacent imide carbonyl, consistent with a local, through‐bond transition.

In contrast, the ISSDA‐based model shown in Figure 3d exhibits a distinctly different chain conformation. The isosorbide‐derived ISSDA units introduce a bent, sterically constrained, and nonplanar bicyclic structure into the polyimide backbone [25, 29], which interrupts the regular approach of neighboring aromatic/imide units.

The TD‐DFT result in Figure 3e shows that the ISSDA‐based model exhibits an intense transition near 206 nm (f = 1.925), substantially stronger than the corresponding band in the ODPA‐based model. This transition lies far outside the near‐UV/visible range relevant to film transparency; a Gaussian lineshape analysis of the calculated spectrum (FWHM = 8 nm) confirms that its contribution at wavelengths ≥ 250 nm is negligible, more than thirteen linewidths removed from the band center, and it therefore does not affect the near‐UV transparency comparison discussed below. Restricting the comparison to the near‐UV region relevant to visible‐light transparency (λ ≥ 250 nm), the ISSDA‐based model instead shows a comparatively weak and blueshifted band at 262 nm (f = 0.082), roughly half the oscillator strength of the analogous ODPA band and located at a shorter wavelength. This indicates that, within the near‐UV region relevant to transparency, the enhanced transparency of the ISSDA‐containing polyimides arises from a combination of reduced transition intensity and a blueshift of the dominant near‐UV transition, rather than from either effect alone.

The frontier orbital analysis in Figure 3f shows that the HOMO and LUMO of the ISSDA‐based model are spatially adjacent, similar to the ODPA‐based model, rather than separated across the two ISSDA termini as we previously reported using B3LYP/6‐31G(d,p). We attribute the earlier result to the well‐documented failure of semi‐local functionals such as B3LYP in describing states with partial through‐space character, a limitation that is substantially corrected by the range‐separated CAM‐B3LYP functional used here. The calculated HOMO and LUMO energy levels of the ISSDA‐based model are −8.33 and −1.87 eV, respectively, giving a HOMO–LUMO gap of 6.46 eV, comparable in magnitude to that of the ODPA‐based model (6.81 eV).

The molecular‐level interpretation was further corroborated by WAXD analysis. As shown in Figure 3g, all polyimide films exhibited broad diffraction features rather than sharp crystalline peaks, indicating predominantly amorphous structures [27]. The main amorphous halo gradually shifted toward larger d‐spacing as ISSDA content increased, from the ODPA‐only film at smaller d‐spacing to the ISSDA‐rich composition at approximately 5–6 Å. This trend experimentally confirms that ISSDA incorporation expands the average interchain distance and disrupts compact chain packing in the bulk polymer, and provides the primary quantitative evidence for the interchain packing argument [25, 27].

The two‐dimensional WAXD pattern of the optimized film in Figure 3h also showed an isotropic amorphous halo without distinct crystalline reflections. This isotropic amorphous structure is advantageous for transparent polymer films because it minimizes light scattering caused by ordered domains or crystalline aggregates [16, 17], and is expected to facilitate local chain rearrangement during thermal healing, linking the optical transparency mechanism to the rapid healing behavior discussed in the following sections [1, 14].

Taken together, Figure 3 demonstrates that the enhanced transparency of the ISSDA‐containing polyimides arises from a combined electronic and packing effect: at the single‐chain level, the nonplanar ISSDA structure suppresses and blueshifts the near‐UV electronic transitions relevant to transparency; at the supramolecular level, confirmed experimentally by WAXD, ISSDA enlarges interchain d‐spacing and promotes amorphous packing. The single‐chain DFT/TD‐DFT results describe intramolecular electronic structure only; the interchain packing argument itself rests on the experimental WAXD data rather than on any dimer‐based computational model.

### 2.4 Polymer Formation and Molecular‐Weight Characteristics

Tables 1 and 2 summarize the molecular‐weight characteristics of the copolyimides prepared with different dianhydride and diamine compositions, respectively. Overall, all compositions produced film‐forming polyimides, confirming that the aliphatic–aromatic hybrid polymerization strategy adopted in this study is broadly applicable across the investigated compositional range. In particular, even the ISSDA‐containing systems afforded sufficiently high molecular weights for the formation of self‐supporting films, indicating that the introduction of the sterically bulky and nonplanar bio‐based dianhydride did not prevent successful polyimide formation.

**TABLE 1:** Molecular weight and PDI of polyimides with different ISSDA:ODPA molar ratios.

Dianhydride molar ratio (ISSDA:ODPA)	Diamine molar ratio (Cys:DDA)	Total molar ratio (Dianhydride/Diamine)	Molecular Weight (10^5 ^g/mol)	PDI
0:10	5:5	1:1	2.9	1.23
2:8			2.0	1.09
4:6			1.7	1.10
6:4			1.3	1.07

**TABLE 2:** Molecular weight and PDI of polyimides with different Cys:DDA molar ratios.

Diamine Molar ratio (Cys:DDA)	Dianhydride Molar ratio (ISSDA:ODPA)	Total molar ratio (Dianhydride/Diamine)	Molecular Weight (10^5 ^g/mol)	PDI
1:9	6:4	1:1	2.5	1.23
2:8			2.1	1.11
3:7			1.9	1.14
4:6			1.5	1.22
5:5			1.3	1.07

For the ISSDA:ODPA series (Table 1), a decreasing trend in molecular weight was observed with increasing ISSDA content. This trend is reasonable considering that ISSDA possesses a contorted three‐dimensional structure that may increase steric hindrance during chain growth. Thus, while ISSDA is highly beneficial for suppressing dense chain packing and improving transparency, excessive incorporation may also impose limitations on molecular‐weight buildup and may affect the balance among chain rigidity, processability, and film formation. In this regard, the optimization of the ISSDA:ODPA ratio should be understood not only as a transparency‐control strategy, but also as a synthetic design parameter that governs polymerizability and the accessible molecular‐weight range.

A parallel trend was observed in the Cys:DDA series (Table 2), where molecular weight decreased as Cys content increased. Although this series varies the diamine component rather than the dianhydride, we note that in both series the molecular‐weight decrease coincides with replacing a more extended, conformationally flexible monomer (ODPA in Table 1; DDA in Table 2) with a more compact, structurally constrained one (ISSDA in Table 1; Cys in Table 2), suggesting that steric or conformational constraints on chain‐extension efficiency during step‐growth polymerization operate similarly regardless of whether the constraining unit originates from the dianhydride or the diamine component. Because Cys and DDA play fundamentally different roles in the present system, this trend is also important for excluding the possibility that the observed differences in healing or mechanical performance arise merely from polymerization failure, rather than indicating that these differences are primarily associated with composition‐dependent structural effects.

It is noteworthy that the optimized composition did not necessarily correspond to the sample with the highest molecular weight. This observation suggests that the superior combination of transparency, healing performance, and toughness in the present system cannot be attributed simply to maximized molecular weight. Rather, it appears to arise from a favorable balance among several composition‐dependent structural factors, including free‐volume‐rich chain packing, segmental mobility, and dynamic bond exchange capability. In other words, once a sufficient molecular‐weight window for film formation is achieved, the dominant factors controlling the final performance are the monomer‐derived structure–property relationships rather than molecular weight alone.

From this perspective, Tables 1 and 2 provide an important foundation for interpreting the later property results. ISSDA contributes to transparency and free‐volume generation but may influence steric accessibility during polymerization; Cys provides dynamic covalent functionality; DDA enhances chain mobility and low‐temperature healing capability. The fact that all of these components can be incorporated while maintaining a practical molecular‐weight range demonstrates that the present system is not based on a fortuitous single composition, but on a synthetically viable molecular platform capable of integrating optical, mechanical, and healing‐related functions. The potential role of decreasing Mw as an internal plasticizer is examined quantitatively in Section 2.5.

### 2.5 Composition‐Dependent Glass Transition and Healing Behavior

Figure 4 compares the glass‐transition behavior, healing efficiency, and crack‐closure kinetics of the present polyimides as a function of monomer composition. These data clearly show that the self‐healing performance of the system is governed not solely by the presence of disulfide chemistry, but by the interplay among thermal mobility, free‐volume‐rich packing, and dynamic bond exchange.

**FIGURE 4:**
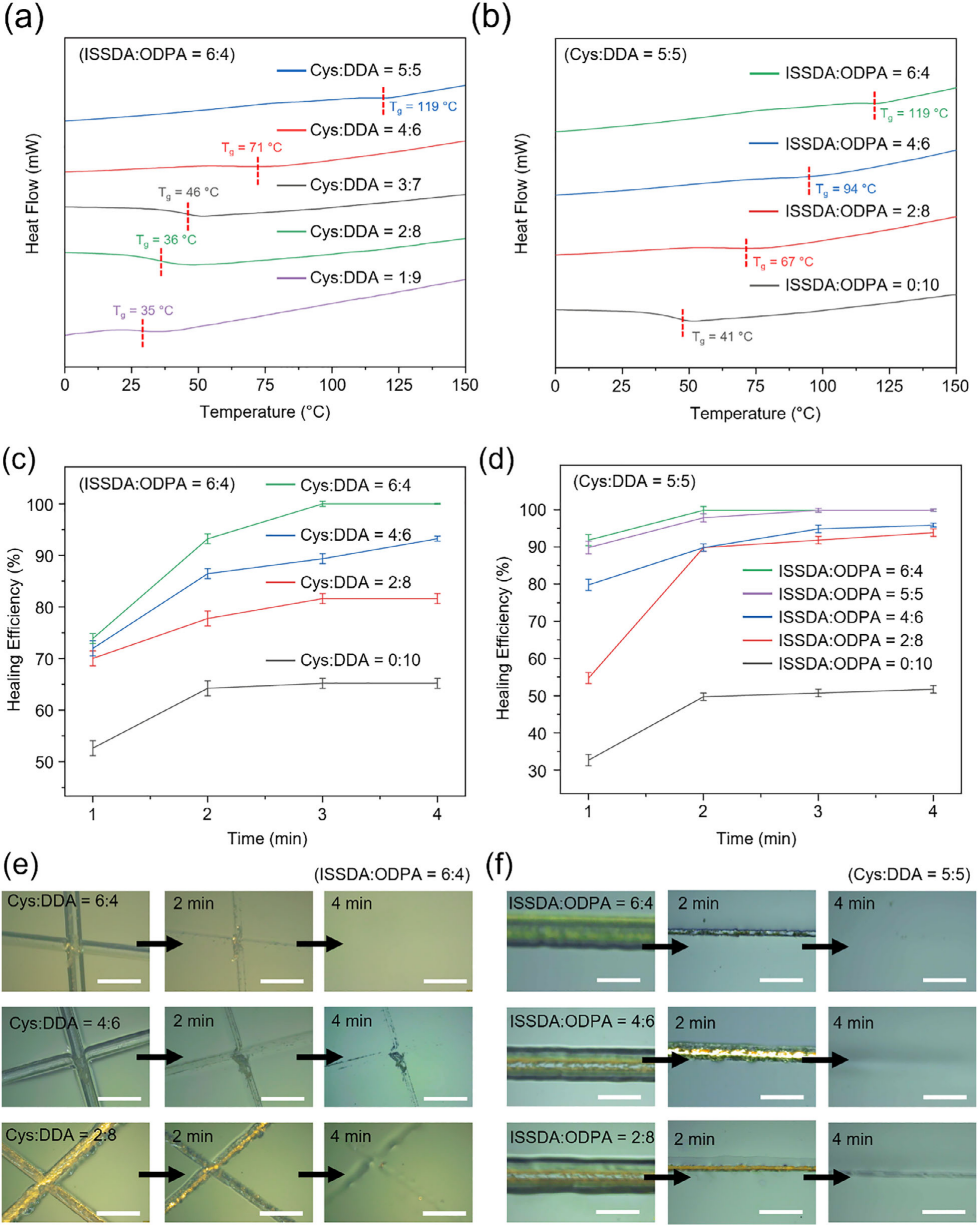
Composition‐dependent glass transition temperature and healing behavior. (a) DSC curves of polyimide films with varying Cys:DDA molar ratios. (b) DSC curves of polyimide films with varying ISSDA:ODPA molar ratios. (c, d) Crack‐width‐based (optical) healing efficiency of polyimide films with different monomer compositions as a function of healing time, measured at temperatures at least 30°C above Tg. Error bars represent the standard deviation from n ≥ 5 independent measurements. (e, f) Time‐dependent optical microscopy images of crack closure for polyimide films with different monomer compositions during healing performed at temperatures at least 30°C above Tg.

Figure 4a presents the DSC thermograms for the Cys:DDA series. As the DDA content increased, the glass‐transition temperature gradually decreased, reaching values as low as approximately 35°C. This result indicates that the long aliphatic chain of DDA effectively increases segmental flexibility and lowers the thermal barrier for chain rearrangement. Because self‐healing in polymeric systems typically requires sufficient local chain mobility in addition to reversible or exchangeable bonding motifs [1, 14], the decrease in Tg induced by DDA is highly significant. It indicates that DDA functions not merely as a soft segment, but as a monomeric design element that broadens the accessible healing‐temperature window and promotes healing under comparatively mild thermal conditions [2].

In contrast, the ISSDA:ODPA series in Figure 4b exhibited Tg values over a wider range of approximately 41°C–119°C, reflecting the combined effects of chain rigidity and packing structure. The incorporation of ISSDA introduces a rigid yet nonplanar dianhydride unit, which increases structural stiffness while simultaneously suppressing overly dense packing through its contorted geometry. Therefore, ISSDA plays a dual role: it influences the thermal stiffness of the polymer backbone, while also modulating the free‐volume environment in which chain rearrangement and healing occur. Notably, this trend argues against a simple Mw‐plasticization effect. According to the Fox–Flory relation (T_g_ = T_g,∞_ − K/Mn), a lower Mw should lower T_g_ through increased chain‐end free volume. However, the opposite trend is observed. As Mw decreases from 2.9 × 10^5^ to 1.3 × 10^5^ g/mol with increasing ISSDA content, Tg instead rises from 41°C to 119°C. This indicates that the backbone‐rigidifying effect of ISSDA dominates over the Mw/free‐volume effect. Mw is nonetheless expected to independently affect chain diffusion through entanglement density. To properly account for dispersity, we use Mn ( = Mw/PDI) rather than Mw. The ∼1.9‐fold decrease in Mn corresponds to an estimated 7‐ to 10‐fold decrease in reptation time (τ_d_ ∝ M^3–3.4^). A shorter reptation time facilitates faster interfacial chain interdiffusion during healing. This physical contribution is independent of the ISSDA‐ and Cys‐derived effects. We therefore attribute enhanced healing performance to three distinct, cooperating contributions rather than to molecular design alone: (i) ISSDA‐induced free volume generation and CTC suppression, (ii) a Mw‐dependent reduction in entanglement density, and (iii) Cys‐mediated dynamic disulfide exchange.

The healing temperature for each composition was normalized to Tg + 30°C. We did not use a single fixed absolute temperature for all compositions. This normalization allows differences in healing performance to reflect composition‐dependent molecular design. Otherwise, the result would simply reflect how far above Tg each sample was tested. According to the Williams–Landel–Ferry (WLF) free‐volume framework, materials compared at the same T − Tg offset occupy a similar free‐volume state. This holds regardless of their absolute Tg values. The healing‐efficiency plots in Figure 4c,d show that healing response still depends strongly on composition, even under this normalized comparison. Figure 4c compares the Cys:DDA series, revealing that DDA‐rich compositions exhibited substantially faster healing kinetics and higher early‐stage healing efficiencies. By contrast, compositions with excessive Cys content showed slower recovery despite their higher density of disulfide bonds. This result highlights an important point: increasing the concentration of dynamic bonds alone does not necessarily maximize healing efficiency [1, 3, 18]. Instead, effective healing requires that the exchangeable bonds operate within a matrix that possesses sufficient segmental mobility and interfacial accessibility. The detailed procedure used to calculate healing efficiency is provided in Supporting Information. It should be emphasized that the healing efficiency values in Figure 4c,d quantify the optical/geometric closure of the damaged region, rather than the recovery of bulk mechanical properties; a complementary mechanical assessment is presented in Section 2.6.

Figure 4d shows the healing behavior of the ISSDA:ODPA series. The healing efficiency increased more rapidly for compositions containing an appropriate fraction of ISSDA, and the optimized composition reached the highest recovery within a relatively short healing time. If healing were governed only by disulfide exchange density, similar recovery behavior might be expected once the samples were heated above Tg. However, the observed dependence on ISSDA content indicates that the local structural environment also plays a decisive role. The bulky and nonplanar ISSDA unit is considered to promote healing by increasing free volume, loosening chain packing, and facilitating interfacial chain contact and rearrangement at the damaged region.

This interpretation is supported by the optical microscopy images in Figure 4e,f, which directly visualize the time‐dependent crack‐closure process. In Figure 4e, the Cys:DDA series shows that crack disappearance proceeded more rapidly when sufficient DDA was present, consistent with the enhanced chain mobility inferred from the Tg results. Similarly, Figure 4f shows that the ISSDA‐containing compositions exhibited faster and more uniform crack closure than the ODPA‐rich counterparts. These microscopy results are in excellent agreement with the healing‐efficiency data and confirm that the healing process is a kinetic sequence involving interfacial contact, local diffusion, chain rearrangement, and dynamic bond exchange, rather than a purely thermodynamic phenomenon.

Taken together, Figure 4 demonstrates that the superior healing performance of the present system originates from the cooperative optimization of three factors: DDA lowers Tg and promotes segmental mobility, ISSDA provides a free‐volume‐rich and less densely packed chain environment, and Cys supplies the dynamic disulfide exchange chemistry necessary for interfacial rebonding. Therefore, the key to efficient healing in the present polyimide system is not simply the introduction of a dynamic functional group, but the simultaneous control of Tg, chain‐packing structure, and exchangeable bond chemistry through monomer‐level design.

### 2.6 Mechanical Recovery and Toughness Retention After Healing

Figure 5 evaluates the tensile behavior and toughness retention of the present bio‐based aliphatic–aromatic polyimides before and after healing. These results are particularly important because self‐healing polymers often achieve good visual or morphological recovery at the expense of mechanical integrity, and the healed interface frequently remains the weakest point under tensile loading [1, 3, 6, 14, 21, 23, 24, 32, 33]. In contrast, the present system was designed not only to heal rapidly and remain optically transparent, but also to preserve mechanical functionality after damage recovery [34].

**FIGURE 5:**
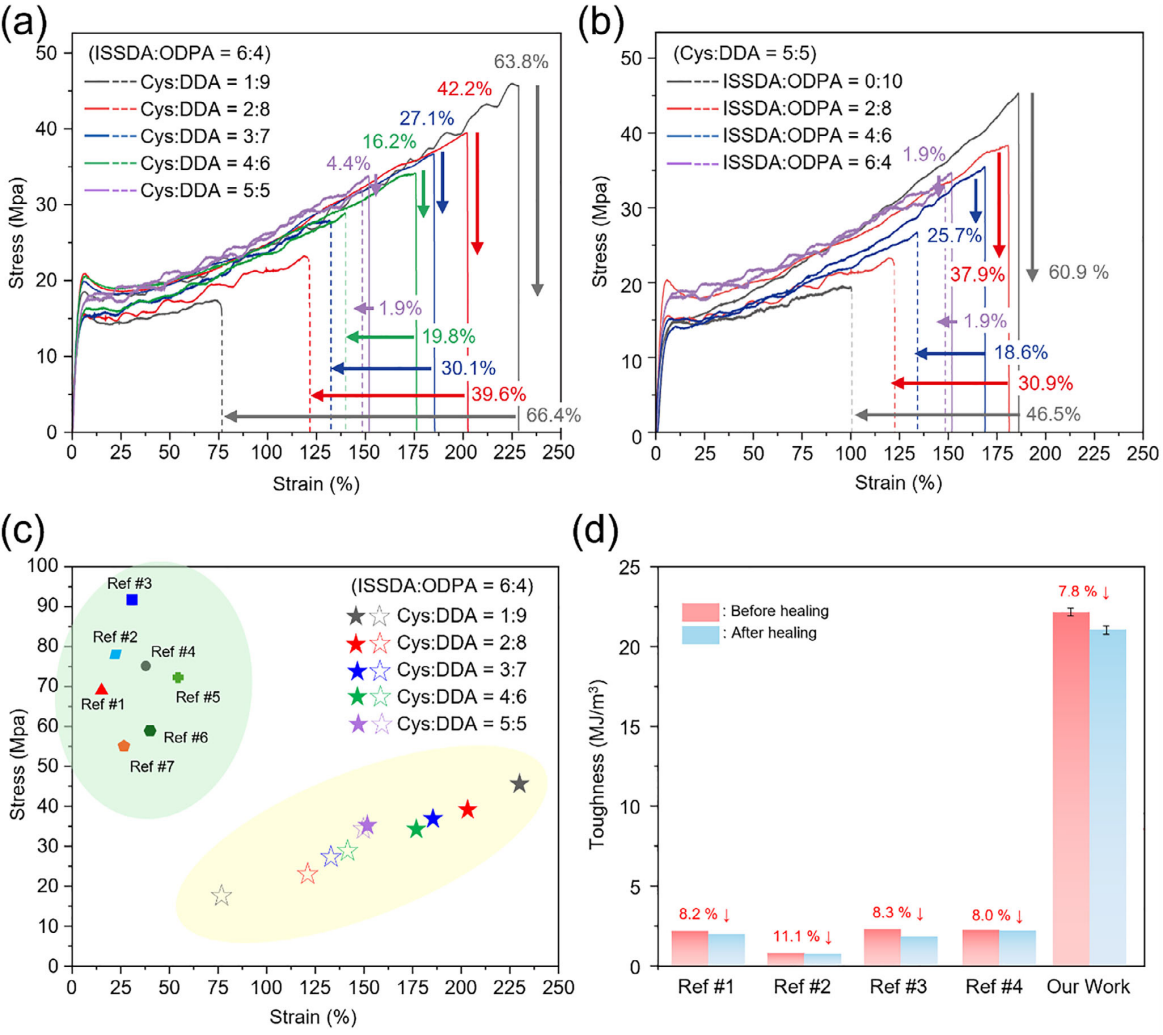
Mechanical property crack recovery before and after healing. (a) Tensile stress–strain curves of polyimide films with varying Cys:DDA molar ratios before and after healing. The inset values indicate the decreases in tensile stress and strain after healing. (b) Tensile stress–strain curves of polyimide films with varying ISSDA:ODPA molar ratios before and after healing. The inset values indicate the decreases in tensile stress and strain after healing. (c) Comparison map of tensile strength and elongation at break, highlighting the superior balance of toughness and extensibility achieved in this work relative to previously reported self‐healable transparent polyimides. Filled symbols represent the break‐point values before healing, whereas open symbols represent those after healing. Notably, for the optimized composition, ISSDA:ODPA = 6:4 and Cys:DDA = 5:5, the symbols nearly overlap, indicating negligible changes in tensile properties after healing. (d) Toughness of representative polyimide films (ISSDA:ODPA = 6:4 and Cys:DDA = 5:5) before and after healing, showing only minor reductions; error bars represent the standard deviation from n ≥ 5 independent measurements. The decrease in toughness was also compared with those of previous works, confirming the smallest loss upon healing in the present system; literature values (Ref #1–4) are plotted as originally reported, without error bars, as replicate data were not available from the source publications.

Figure 5a shows the tensile stress–strain behavior of the Cys:DDA series before and after healing. As the DDA content increased, the films generally exhibited improved elongation, and the loss in tensile performance after healing became less pronounced. This trend is consistent with the role of DDA as a long‐chain aliphatic diamine that promotes interfacial chain rearrangement and recovery at the damaged region. By contrast, Cys‐rich compositions tended to retain relatively high stiffness but showed more limited recovery in post‐healing deformation behavior. These results again indicate that dynamic bond density alone is not sufficient to ensure effective mechanical recovery; mobility‐assisted interfacial reconstruction is also essential.

A comparable trend was observed in the ISSDA:ODPA series shown in Figure 5b. Compositions containing an appropriate fraction of ISSDA showed only small differences between the tensile curves measured before and after healing, whereas ODPA‐rich films experienced larger losses in stress and strain. This result suggests that ISSDA contributes not only to optical transparency, but also to the formation of a structural environment favorable for post‐damage recovery. The nonplanar and sterically bulky ISSDA unit is considered to facilitate chain interdiffusion and rearrangement across the damaged interface by suppressing overly dense packing and maintaining a free‐volume‐rich local structure. Thus, the same monomeric design principle that improves transparency also appears to benefit mechanical recovery after healing.

The superiority of the present system becomes more evident in the tensile strength–elongation map shown in Figure 5c. Compared with previously reported self‐healable polymer systems, the present compositions occupy a more favorable region that combines relatively high strength with large elongation at break. (Ref #1 [14], Ref #2 [31], Ref #3 [32], Ref #4 [34], Ref #5 [6], Ref #6 [1], Ref #7 [3]). More importantly, for the optimized composition (ISSDA:ODPA = 6:4 and Cys:DDA = 5:5), the break‐point values before and after healing nearly overlap. This indicates that the healed specimen retained mechanical performance very close to that of the original film. Such behavior is highly significant, because in many self‐healing polymers a nominally high healing efficiency does not necessarily translate into recovery of the original break‐point behavior or load‐bearing capability [6, 14].

Figure 5d further compares the toughness of the representative optimized film before and after healing, together with values from previously reported systems. The optimized composition exhibited only a minor reduction in toughness after healing, approximately 7.8%, which is smaller than or comparable to the losses reported in earlier studies while maintaining markedly higher absolute toughness. (Ref #1 [31], Ref #2 [32], Ref #3 [3], Ref #4 [14]) Expressed as a mechanical healing efficiency, η_mechanical_ = (toughness after healing / original toughness) × 100%, this corresponds to ≈92.2% for the optimized composition, providing a property‐based counterpart to the optical healing efficiency reported in Figure 4. Because toughness reflects the combined contributions of strength and elongation, this small post‐healing reduction indicates that the healed interface can still absorb mechanical energy in a manner similar to the original material. In other words, the recovered interface is not merely reattached, but remains mechanically functional under tensile deformation.

Importantly, this outstanding toughness cannot be interpreted simply as a molecular‐weight effect, because the optimized composition does not correspond to the highest‐Mw sample in the series. As discussed quantitatively in Section 2.5, this cannot be attributed to Mw‐driven plasticization alone, since the glass‐transition trend argues against it; rather, entanglement‐density effects and ISSDA/Cys‐mediated structural and chemical contributions act cooperatively. The present results suggest that the mechanical superiority of the optimized system arises from a favorable structural balance established once a sufficient molecular‐weight range for robust film formation is secured. In the present case, several factors appear to contribute cooperatively. First, the polyimide backbone still retains sufficient cohesive interaction through the imide‐containing hard segments, preventing excessive softening despite the incorporation of flexible aliphatic chains. Second, the long‐chain DDA units provide soft‐segment‐like flexibility and enhanced deformability, which improve elongation and facilitate stress dissipation under tensile loading. Third, the disulfide‐containing Cys segments enable dynamic bond exchange and interfacial rebonding after damage. Fourth, the incorporation of ISSDA generates a less densely packed and more free‐volume‐rich chain environment, which can promote chain interpenetration and rearrangement across the healed interface without completely destroying structural integrity.

From this perspective, the present system may be regarded as a compositionally balanced hard/soft polyimide network rather than a polymer whose performance is dictated by molecular weight alone. Although no permanent covalent cross‐linking was intentionally introduced, the combination of rigid imide segments, contorted ISSDA units, long aliphatic DDA chains, and dynamic disulfide bonds appears to create an effectively integrated mechanical framework in which cohesive interaction, segmental mobility, and reversible interfacial reconstruction are simultaneously optimized. In other words, the high toughness of the optimized composition likely originates not from a single dominant factor, but from the cooperative action of backbone rigidity, local flexibility, dynamic exchangeability, and structurally moderated chain packing. This interpretation also explains why high toughness, efficient healing, and transparency could be achieved simultaneously even though the molecular weight was not maximized.

Overall, Figure 5 provides strong evidence that the present molecular design overcomes one of the most persistent limitations in transparent self‐healing polyimides: the trade‐off among transparency, healing efficiency, and mechanical robustness. In the optimized composition, the films not only heal rapidly and remain highly transparent, but also retain tensile properties and toughness to an extent rarely achieved in previously reported systems. This result highlights the value of combining ISSDA and DDA as complementary structural modules for constructing mechanically reliable transparent self‐healing polyimides.

### 2.7 Demonstration of Electrical Function Recovery

Figure 6 presents a proof‐of‐concept demonstration showing that the optimized self‐healable polyimide can also function as a platform for electrical recovery when combined with a conductive network. Based on the preceding results, the composition with ISSDA:ODPA = 6:4 and Cys:DDA = 5:5 exhibited the most favorable balance of transparency, healing kinetics, and mechanical recovery. This optimized film was therefore selected as the substrate for constructing a simple conductive path using a silver nanowire network. The silver nanowires used in this study had an average diameter of approximately 300 nm and an average length of approximately 15 µm, and their SEM images are provided in Figure S7.

**FIGURE 6:**
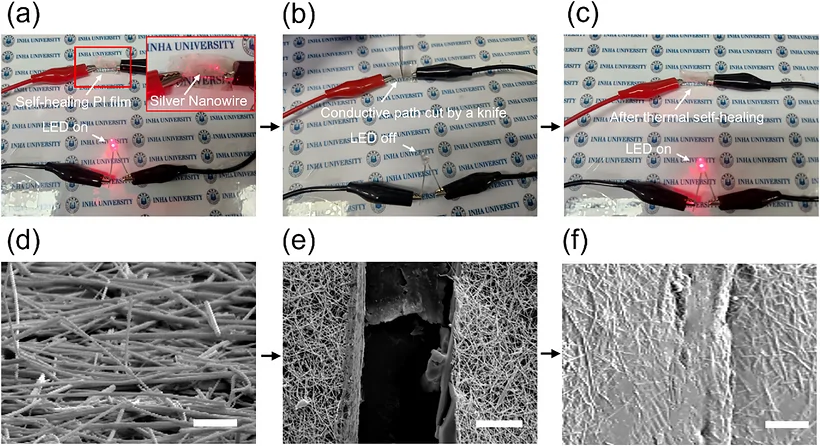
Electrical self‐healing demonstration. (a) Optical images of the silver nanowire‐coated self‐healable polyimide film in the initial state, showing stable electrical conduction with the LED on. The inset presents a magnified view of the conductive region. (b) Optical images after cutting the conductive path with a knife, resulting in loss of electrical conduction and LED turn‐off. (c) Optical images after thermal self‐healing, showing restoration of the conductive path and recovery of LED operation. (d) SEM image of the silver nanowire network formed on the polyimide surface. (scale bar: 2 µm) (e) SEM image of the damaged region after cutting. (scale bar: 10 µm) (f) SEM image of the healed region after thermal self‐healing. (scale bar: 5 µm).

As shown in Figure 6a, the silver nanowire‐coated self‐healable PI film exhibited stable electrical conduction in the initial state, as evidenced by illumination of the LED. The inset image further shows that the conductive region remained optically accessible, consistent with the high transparency of the underlying film. This result indicates that the present polyimide is not only a mechanically healable film, but also a promising substrate or coating material for transparent and flexible functional systems.

When the conductive path was severed by knife cutting, the LED was immediately turned off, as shown in Figure 6b, confirming complete loss of electrical continuity. This step is important because it establishes that the mechanical damage directly translated into functional failure of the conductive pathway, thereby allowing the subsequent healing process to be evaluated in terms of functional recovery rather than mere visual restoration.

After thermal healing, the LED was illuminated again, as shown in Figure 6c, indicating recovery of electrical conduction across the previously damaged region. Although this experiment is intended as a qualitative demonstration rather than a quantitative conductivity analysis, it clearly suggests that the present self‐healing PI can support restoration of electrical function when integrated with a conductive nanowire network [35, 36, 37, 38]. In this sense, the role of the polyimide extends beyond structural healing and into the realm of functional materials platforms for flexible electronics [18, 39, 40, 41, 42].

The SEM images in Figures 6d–f provide microstructural support for this interpretation. Figure 6d shows a relatively dense and continuous silver nanowire network formed on the PI surface, which is favorable for initial electrical percolation. Figure 6e reveals a distinct gap and disruption of the conductive layer after cutting, whereas Figure 6f shows that the damaged region became substantially reclosed after thermal healing. These observations are consistent with the LED on/off/on sequence and suggest that the restoration of macroscopic electrical function is closely related to the reestablishment of interfacial contact and local conductive continuity at the healed region.

Taken together, Figure 6 demonstrates that the present optimized self‐healing PI is not limited to passive structural recovery, but can also serve as a functional platform for simple electrical restoration when combined with a conductive network [18]. Although this demonstration does not yet represent a fully quantified device study, it provides an important proof‐of‐concept for extending the present material system toward transparent flexible electronics, self‐healable conductive substrates, and protective films with recoverable functionality [23, 35, 43, 44, 45, 46, 47, 48].

## 3 Conclusion

A bio‐based aliphatic–aromatic self‐healable polyimide platform was successfully developed by integrating ISSDA, ODPA, cystamine, and a bio‐based long‐chain aliphatic diamine (DDA). The combined use of ISSDA and DDA enabled simultaneous control of optical transparency, chain mobility, and healing behavior, thereby alleviating the typical trade‐off among transparency, self‐healing, and mechanical robustness. The optimized composition (ISSDA:ODPA = 6:4, Cys:DDA = 5:5) showed approximately 88% transmittance at 550 nm and over 95% transmittance above 650 nm, rapid thermal healing at 150°C within 4 min, and excellent toughness retention with only a 7.8% reduction after healing. Structural analyses further revealed that ISSDA suppresses and blueshifts the near‐UV electronic transitions relevant to transparency and enlarges interchain spacing while promoting amorphous packing, while the long‐chain aliphatic character of DDA and the dynamic disulfide exchange of Cys contributed to efficient interfacial recovery. A proof‐of‐concept demonstration further showed that the optimized film can support restoration of electrical function when integrated with a silver nanowire conductive network, suggesting its applicability as a transparent and self‐healable substrate for flexible electronics. These findings provide a practical molecular design strategy for sustainable transparent self‐healable engineering polyimides and suggest their potential in flexible electronic and functional coating applications.

## 4 Experimental Section

### 4.1 Materials

Isosorbide (> 98.0%), anhydrous trimellitic acid chloride (> 98.0%), and cystamine dihydrochloride (>97.0%) were purchased from TCI (Japan). Acetonitrile (> 99.5%), acetic anhydride (> 97%), dichloromethane (DCM, 99.5%), and *N,N*‐dimethylacetamide (DMAc) were obtained from Daejung Chemicals (Korea). Pyridine (> 99.0%) was purchased from Samchun Chemicals (Korea). Potassium hydroxide and calcium chloride were obtained from Duksan Chemicals (Korea). 4,4′‐Oxydiphthalic anhydride (ODPA, > 98%) was purchased from TCI (Japan). Priamine 1075 was supplied by Croda/Cargill (USA). All chemicals were used as received unless otherwise noted.

### 4.2 Synthesis of ISSDA

Anhydrous trimellitic acid chloride (5.91 g, 0.028 mol) was dissolved in acetonitrile (10 mL) in a three‐neck flask under a nitrogen atmosphere and cooled to 0°C–5°C. In a separate vessel, isosorbide (2.0 g, 0.014 mol) and pyridine (3.25 g, 0.041 mol) were dissolved in acetonitrile (12 mL). The isosorbide solution was added dropwise to the acid chloride solution over 1 h using a dropping funnel while maintaining the internal temperature at 0°C–5°C. After complete addition, the mixture was stirred at 0°C–5°C for 1 h and then at room temperature overnight. The white precipitate was collected by filtration and recrystallized from acetic anhydride. The product was dried in a vacuum oven at 70°C overnight.

### 4.3 Synthesis of Cystamine

Cystamine dihydrochloride (16 g, 71 mmol) was dissolved in distilled water (50 mL) with stirring for 30 min. Separately, potassium hydroxide (12 g, 213 mmol, 3.0 equiv) was dissolved in distilled water (50 mL). The KOH solution was slowly added to the cystamine solution and stirred at room temperature for 30 min. The mixture was extracted with dichloromethane (200 mL total, three times). The organic phase was dried over anhydrous CaCl_2_ (10 g), filtered, and concentrated under reduced pressure to yield cystamine as a pale‐yellow oily liquid.

### 4.4 Synthesis of Self‐Healable Polyimide (SHPI)

Cystamine (0.005 mol) and Priamine 1075 (0.005 mol) were dissolved in DMAc in a three‐neck flask with stirring at room temperature for 2 h. ISSDA (0.006 mol) and ODPA (0.004 mol) were then added in three portions, and the mixture was stirred at 60°C for 24 h to obtain a poly(amic acid) (PAA) precursor solution. The total monomer concentration in DMAc was approximately 15 wt%. The PAA solution was cast onto a glass Petri dish and degassed under vacuum at room temperature for 50 min. Thermal imidization was carried out under vacuum at 70°C for 2 h, 100°C for 1 h, 150°C for 1 h, and 200°C for 1 h. After imidization, the film was immersed in warm deionized water overnight and peeled off to yield a free‐standing self‐healable polyimide film.

### 4.5 Computational Analysis of Molecular Structure and Electronic Transitions

Density functional theory (DFT) and time‐dependent DFT (TD‐DFT) calculations were performed using Gaussian 16. Symmetric, single‐chain ODPA‐ and ISSDA‐based polyimide model structures (ODPA‐Cys‐ODPA‐DDA and ISSDA‐Cys‐ISSDA‐DDA, respectively) were constructed, each containing two dianhydride units, one Cys unit, and one DDA unit in an identical sequence and differing only in the dianhydride identity. Because Priamine 1075 (DDA) is a technical‐grade mixture of branched C36 isomers without a single well‐defined structure, its aliphatic segment was represented in both models by a simplified branched fragment. Geometry optimizations were carried out at the B3LYP/6‐311+G(2d,p) level without symmetry constraints, followed by frequency calculations to confirm that the optimized structures corresponded to true local minima (no imaginary frequencies). Frontier molecular orbital distributions, HOMO and LUMO energy levels, and HOMO–LUMO energy gaps were obtained from the optimized single‐chain structures. TD‐DFT calculations were performed at the CAM‐B3LYP/6‐311G(d,p) level (fifty lowest singlet excited states) to obtain the simulated UV–vis absorption profiles, molar extinction coefficients, and oscillator strengths; no interchain distance was calculated from these single‐chain models. The calculated transition intensity was used as a qualitative indicator of CTC‐related optical absorption rather than as an absolute quantitative prediction of film transmittance. Further computational details are provided in Section S9.

### 4.6 Characterization

FT–IR spectra were recorded using a Nicolet 5700 spectrometer (Thermo Fisher Scientific, USA) in the range of 600–4000 cm^−^^1^. ^1^H NMR spectra were recorded on a Bruker Avance Neo 600 MHz spectrometer in DMSO‐d_6_. Molecular weights and PDIs were determined by gel permeation chromatography (GPC; Waters, USA) using DMF containing 0.05 M LiBr as the eluent at a flow rate of 1.0 mL min^−^^1^ at 40°C, calibrated with polystyrene standards. UV–vis transmittance spectra were recorded on a UV–vis spectrophotometer (Shimadzu UV‐2600, Japan) using free‐standing films of approximately 50 µm thickness. DSC was performed under a nitrogen atmosphere over the range of −60°C to 250°C at a heating rate of 10°C min^−^^1^; Tg values were taken from the second heating scan. WAXD analysis was conducted using a Rigaku SmartLab diffractometer with Cu Kα radiation (λ = 1.5406 Å) over a 2θ range of 5°–40°, and two‐dimensional WAXD patterns were collected to confirm the amorphous nature of the optimized film. Tensile properties were measured using a universal testing machine (UTM; Instron 5966, USA) at a crosshead speed of 10 mm min^−^^1^ on dog‐bone‐shaped specimens with a gauge length of 25 mm and width of 5 mm; tensile strength, elongation at break, and toughness were obtained from the stress–strain curves before and after healing. For healing experiments, damaged specimens were placed on a hot stage and thermally treated at the designated temperature without applied pressure. Optical microscopy was used to monitor crack closure during healing. SEM was performed using a Tescan Vega2 microscope (Tescan, Czech Republic) at an accelerating voltage of 20 kV. Electrical conduction recovery was evaluated qualitatively by monitoring LED illumination before cutting, after cutting, and after thermal healing of the silver nanowire‐coated polyimide film.

## Author Contributions

This manuscript has been prepared with contributions from all authors, who have also approved the final version of the manuscript.

## Conflicts of Interest

The authors declare no conflicts of interest.

## Supporting information




**Supporting File**: smll75559‐sup‐0001‐SuppMat.docx.

## Data Availability

The data that support the findings of this study are available from the corresponding author upon reasonable request.
