# Supplementary material for: Bio‐Based Transparent Self‐Healable Polyimides Enabled by Isosorbide‐Derived Dianhydride Design and Aliphatic Chain‐Mobility Regulation
Source: Small. 2026 Sep 3;22(62):e75559. doi: 10.1002/smll.75559 (PMC13742889; doi:10.1002/smll.75559)
Supplement: Supplementary file 1 [file SMLL-22-e75559-s001.docx]

**Supporting Information**

**Bio-Based Transparent Self-Healable Polyimides Enabled by Isosorbide-Derived Dianhydride Design and Aliphatic Chain-Mobility Regulation**

Jisu Jang^a,+^, Woojin Kang^a,+^, Yeongseok Seo^a^, Gyu Hyoung Bae^a^, Jeong Yeon Yoo^a^, Sang Goo Lee^b^*, Keun-Hwan Oh^c^*, and Hong Suk Kang^a^*

^a^ *Department of Polymer Science and Engineering and Program in Environmental and Polymer Engineering, Inha University, Incheon 22212, Republic of Korea*

^b^*Interface Material and Chemical Engineering Research Center, Korea Research Institute of Chemical Technology, 141 Gajeong-ro, Yuseong-gu, Daejeon 34114, Republic of Korea*

^c^ *Hydrogen Energy Research Center, Korea Research Institute of Chemical Technology, 141 Gajeong-ro, Yuseong-gu, Daejeon 34114, Republic of Korea*

^+^These authors contributed equally to this work

**Corresponding author**: Sang-Goo Lee ([sgoo@krict.re.kr](mailto:sgoo@krict.re.kr)), Keun-Hwan Oh ([khoh@krict.re.kr](mailto:khoh@krict.re.kr)), and Hong Suk Kang ([hskang@inha.ac.kr](mailto:hskang@inha.ac.kr))

**S1. Preparation and Characterization of Isosorbide-Based Dianhydride (ISSDA)**

Following the synthetic route established by Sawada and Ando,^S1^ ISSDA was synthesized through an acylation reaction between isosorbide and anhydrous trimellitic acid chloride, as illustrated in Figure S1. The characterization below confirms the identity and purity of the batch used in this study. All reagents were used as received without further purification: isosorbide (>98.0%, TCI, Japan), anhydrous trimellitic acid chloride (>98.0%, TCI, Japan), acetonitrile (>99.5%, Daejung Chemicals, Korea), pyridine (>99.0%, Samchun Chemicals, Korea), and acetic anhydride (>97%, Daejung Chemicals, Korea).

In a typical procedure, anhydrous trimellitic acid chloride (5.91 g, 0.028 mol) was dissolved in acetonitrile (10 mL) in a three-neck flask equipped with a nitrogen inlet, a mechanical stirrer, and a dropping funnel. The flask was immersed in an ice bath and the internal temperature was carefully maintained at 0–5 °C throughout the addition step. In a separate vessel, isosorbide (2.0 g, 0.014 mol) and pyridine (3.25 g, 0.042 mol) were dissolved in acetonitrile (12 mL) to prepare the diol solution. Pyridine was employed both as a base to neutralize the hydrogen chloride generated during the acylation reaction and as a nucleophilic catalyst to promote the esterification. The isosorbide–pyridine solution was then transferred to a dropping funnel and added dropwise to the trimellitic acid chloride solution over 1 h while maintaining the internal temperature at 0–5 °C. Strict temperature control during addition was essential to suppress side reactions and prevent premature ring closure or anhydride hydrolysis. After the addition was complete, the reaction mixture was stirred at 0–5 °C for an additional 1 h to ensure complete conversion of the acid chloride groups, and then allowed to warm to room temperature and stirred overnight.

The white precipitate formed during the reaction was collected by vacuum filtration. To remove residual pyridinium hydrochloride salt and other impurities, the crude product was recrystallized from acetic anhydride. The use of acetic anhydride as the recrystallization solvent is particularly appropriate for dianhydride purification, as it simultaneously promotes anhydride ring closure of any residual amic acid intermediates while effectively dissolving ionic byproducts. The purified product was subsequently dried in a vacuum oven at 70 °C overnight to yield ISSDA as a white crystalline solid.

The reaction stoichiometry was designed with a slight excess of trimellitic acid chloride (2.0 equiv relative to isosorbide) to ensure complete bifunctional esterification of both hydroxyl groups of isosorbide, thereby minimizing mono-substituted byproduct formation. The resulting ISSDA monomer incorporates the rigid isosorbide bicyclic acetal/ether core, which imparts a non-planar, V-shaped three-dimensional conformation to the dianhydride unit. This contorted geometry is fundamentally distinct from that of conventional planar aromatic dianhydrides and is responsible for the enlarged free volume and suppressed charge-transfer complex formation observed in the resulting polyimides, as discussed in the main text.

**Figure S1.** Synthetic scheme for the preparation of isosorbide-based dianhydride (ISSDA). Step 1: acylation of isosorbide with anhydrous trimellitic acid chloride in the presence of pyridine and acetonitrile at 0–5 °C to afford the diester–diacid intermediate. Step 2: recrystallization from acetic anhydride to yield the final ISSDA product as a white crystalline solid.

**S2. ¹H NMR Characterization of Synthesized ISSDA**

The successful synthesis of ISSDA was further confirmed by ¹H NMR spectroscopy (Figure S2). The spectrum was acquired on a Bruker Avance Neo 600 MHz spectrometer using DMSO-*d*₆ as the deuterated solvent, with tetramethylsilane (TMS) as the internal reference.

The ¹H NMR spectrum exhibited resonance signals fully consistent with the proposed ISSDA structure. In the aromatic region, signals appearing at δ 8.43, 8.17, and 7.92 ppm were assigned to the three inequivalent aromatic protons (H*a*, H*b*, H*c*) of the trimellitic anhydride-derived benzene ring. The asymmetric 1,2,4-trisubstituted substitution pattern of the benzene ring renders all three aromatic protons chemically inequivalent, giving rise to three distinct singlet- or doublet-type signals, which is characteristic of trimellitic anhydride ester derivatives. The observed chemical shifts in the δ 7.9–8.5 ppm region are consistent with aromatic protons adjacent to both the electron-withdrawing anhydride carbonyl and the ester linkage, confirming the formation of the trimellitate moiety.

In the aliphatic region, the isosorbide bicyclic framework gave rise to a series of characteristic signals between δ 3.9 and 6.2 ppm. The bridgehead protons at H-2 and H-5 of the isosorbide core, which adopt fixed *exo* and *endo* orientations due to the rigid fused bicyclic ring geometry, were observed as distinct multiplets at δ 6.12 and 5.98 ppm, respectively. The diastereotopic methine and methylene protons of the isosorbide ring (H-1, H-3, H-4, H-6) appeared as a complex multiplet pattern between δ 3.94 and 4.82 ppm, which is consistent with the stereochemically constrained environment of the isosorbide bicyclic acetal/ether system and in good agreement with previously reported isosorbide-based dianhydride structures.^S1,S2^ Importantly, no residual hydroxyl proton signal was detected in the δ 5.0–5.5 ppm region, confirming complete esterification of both hydroxyl groups of isosorbide. Furthermore, the absence of broad N–H or carboxylic acid signals confirmed that no unreacted acid chloride or ring-opened amic acid impurities were present in the purified product.

The integration ratios of the aromatic protons to the isosorbide ring protons were measured as approximately 6 : 6, consistent with the theoretical ratio expected for the ISSDA structure incorporating two trimellitate units per isosorbide core. The overall spectral pattern, peak multiplicities, and integration ratios collectively confirm the successful preparation of the target ISSDA monomer in high purity. The isolated yield of ISSDA after recrystallization from acetic anhydride was 74%.

**Figure S2.** ¹H NMR spectrum of the synthesized ISSDA (600 MHz, DMSO-*d*₆). Assignments: δ 8.43, 8.17, 7.92 (aromatic H, 6H), 6.12, 5.98 (bridgehead H of isosorbide, 2H), 4.82–3.94 (isosorbide ring H, 6H).

**S3. Physicochemical Characteristics of the Bio-Based Aliphatic Diamine (DDA, Priamine 1075)**

The long-chain aliphatic diamine employed in this study, referred to as DDA, was Priamine 1075 (Croda/Cargill, USA), a commercially available bio-based dimer fatty diamine derived from oleic acid; the specifications below are summarized from the manufacturer's technical data sheet for reference. Unlike ISSDA and cystamine, which were synthesized in-house, DDA was used as received without further purification. The chemical structure and key physicochemical specifications of Priamine 1075 are summarized in Figure S3.

As shown in Figure S3, Priamine 1075 is a C36 dimer diamine produced by reductive amination of dimer fatty acid, itself obtained through thermal oligomerization of C18 unsaturated fatty acids predominantly derived from plant-oil-based oleic acid. The resulting molecular structure consists of a long, branched aliphatic C36 backbone bearing two primary amine (–NH₂) end groups. This architecture is fundamentally distinct from conventional short-chain or aromatic diamines, and it is precisely this extended aliphatic character that enables DDA to function as a soft-segment-like building block in the present polyimide system — enhancing segmental mobility, lowering the effective glass transition temperature, and facilitating interfacial chain rearrangement during thermal self-healing.

The tabulated specifications confirm that Priamine 1075 has an amine value of 205 mgKOH/g and a weight-average molecular weight of 546 g/mol, yielding a theoretical amine equivalent weight of approximately 273 g/mol per –NH₂ group. This value was used as the basis for stoichiometric calculation of the diamine-to-dianhydride molar ratio during polyimide synthesis. The dimer content exceeds 99 wt%, with trimer content below 1 wt%, confirming the bifunctional purity critical for controlled linear step-growth polymerization. The presence of trifunctional trimer species above trace levels would introduce undesired branch points into the polyimide backbone, potentially leading to premature crosslinking and loss of film-forming capability. The confirmed near-unity dimer purity therefore provides a reliable and reproducible synthetic foundation for the preparation of the linear copolyimide chains described in this work. The dynamic viscosity of Priamine 1075 at 25 °C is 210 mPa·s, indicating a liquid state at room temperature that permits direct dissolution in the polymerization solvent (DMAc) without the need for elevated-temperature handling or pre-melting steps.

From a sustainability perspective, the derivation of DDA from oleic acid — a renewable C18 fatty acid predominantly sourced from rapeseed or sunflower oil — qualifies it as a bio-based monomer under standard bio-content classification frameworks. Its incorporation into the present polyimide backbone, alongside the isosorbide-derived ISSDA, contributes to an increased bio-based carbon content in the final material, consistent with the green chemistry design principles underlying this work.

**Figure S3.** Chemical structure and physicochemical specifications of the bio-based aliphatic diamine DDA (Priamine 1075, Croda/Cargill, USA). Specifications include amine value (205 mgKOH/g), weight-average molecular weight (546 g/mol), dimer content (>99 wt%), trimer content (<1 wt%), and dynamic viscosity at 25 °C (210 mPa·s).

**S4. Synthesis and ¹H NMR Characterization of Cystamine**

Following an established procedure,^S3^ cystamine was prepared by deprotonation of cystamine dihydrochloride (CDH) under basic aqueous conditions, as illustrated in the synthetic scheme in Figure S4. The characterization below confirms the identity and purity of the batch used in this study. In a typical procedure, CDH (16 g, 71 mmol) was dissolved in distilled water (50 mL) with stirring for 30 min to prepare a homogeneous aqueous solution. Separately, potassium hydroxide (12 g, 213 mmol, 3.0 equiv) was dissolved in distilled water (50 mL) with stirring for 30 min. The use of 3.0 equivalents of KOH relative to CDH ensures complete neutralization of both hydrochloride salts per cystamine unit (2.0 equiv) with an additional excess to drive the deprotonation equilibrium toward complete liberation of the free diamine. The KOH solution was then slowly added to the CDH solution at room temperature, and the mixture was stirred for an additional 30 min to allow complete neutralization and phase separation of the free-base cystamine.

Following the reaction, cystamine was extracted from the aqueous mixture using dichloromethane (DCM, 200 mL total), repeated three times to ensure sufficient recovery of the product into the organic phase. The combined DCM extracts were dried over anhydrous calcium chloride (CaCl₂, 10 g) to remove residual moisture, and the drying agent was subsequently removed by filtration. The dried DCM solution was concentrated using a rotary evaporator to remove the solvent, yielding cystamine as a pale yellow oily liquid. No further purification was required, as the liquid–liquid extraction process effectively separated the free-base cystamine from the potassium chloride byproduct and residual water.

The chemical identity and purity of the synthesized cystamine were confirmed by ¹H NMR spectroscopy (Figure S4), recorded on a Bruker Avance Neo 600 MHz spectrometer in DMSO-*d*₆. The ¹H NMR spectrum exhibited two characteristic triplet signals, fully consistent with the highly symmetric structure of cystamine (H₂N–CH₂–CH₂–S–S–CH₂–CH₂–NH₂). The signal at δ 3.02 ppm (t, *J* = 6.8 Hz, 4H) was assigned to the methylene protons alpha to the primary amine groups (–*CH₂*–NH₂), and the signal at δ 2.75 ppm (t, *J* = 6.8 Hz, 4H) was assigned to the methylene protons alpha to the disulfide linkage (–*CH₂*–S–S–). The downfield shift of the –CH₂–NH₂ protons relative to the –CH₂–S– protons is consistent with the stronger deshielding effect of the primary amine group compared to the disulfide linkage. The 1:1 integration ratio of the two triplets and the absence of any residual signals attributable to the hydrochloride salt form confirm the high purity of the obtained product and the complete removal of ionic byproducts during workup.

The disulfide bond incorporated into the cystamine unit serves as the key dynamic covalent exchange site within the present polyimide system. Under thermal activation above the glass transition temperature, the disulfide bonds can undergo reversible metathesis exchange reactions at the damaged interface, providing the chemical pathway for self-healing. The use of the free-base form of cystamine, rather than its dihydrochloride salt, is essential for controlled step-growth polymerization, as residual HCl would disrupt the stoichiometric balance between diamine and dianhydride monomers, suppress polymerization, and potentially promote hydrolysis of the anhydride functional groups.

**Figure S4.** Synthetic scheme and ¹H NMR characterization of cystamine (600 MHz, DMSO-*d*₆). The spectrum exhibits two triplets at δ 3.02 ppm (–CH₂–NH₂, 4H) and δ 2.75 ppm (–CH₂–S–S–, 4H), confirming the successful preparation of free-base cystamine from cystamine dihydrochloride.

**S5. Polyimide Film Preparation and FT-IR Confirmation of Imidization**

**Polyimide Film Preparation**

Self-healable polyimide films were prepared via a two-step poly(amic acid) (PAA) formation and subsequent thermal imidization process, as illustrated in the synthetic scheme in Figure S5. In a representative procedure for the optimized composition (ISSDA:ODPA = 6:4, Cys:DDA = 5:5), synthesized cystamine (0.005 mol) and Priamine 1075 (DDA, 0.005 mol) were added to *N,N*-dimethylacetamide (DMAc) in a three-neck flask and stirred at room temperature for 2 h to ensure complete dissolution of both diamine components. Subsequently, ISSDA (0.006 mol) and ODPA (0.004 mol) were added slowly to the diamine solution in three equal portions to prevent localized exothermic heat generation and ensure uniform dissolution of the dianhydride monomers. The reaction mixture was then stirred at 60 °C for 24 h under a nitrogen atmosphere to obtain a viscous poly(amic acid) precursor solution. Other compositions were prepared analogously by varying the molar ratios of ISSDA:ODPA and Cys:DDA while maintaining the total dianhydride-to-diamine molar ratio at 1:1.

The resulting PAA solution was cast onto a clean glass Petri dish and degassed under vacuum at room temperature for 50 min to remove dissolved air bubbles. Thermal imidization was subsequently carried out in a vacuum oven using a stepwise heating program: 70 °C for 2 h, 100 °C for 1 h, 150 °C for 1 h, and 200 °C for 1 h. This stepwise protocol was adopted to allow gradual evaporation of the residual solvent at lower temperatures before promoting the cyclodehydration reaction at higher temperatures, thereby minimizing void formation and film cracking. After completion of the imidization cycle, the resulting polyimide film was immersed in warm deionized water overnight to promote delamination from the glass substrate, and then carefully peeled off to obtain a free-standing, self-supporting polyimide film.

**FT-IR Confirmation of Successful Thermal Imidization**

The successful conversion of the PAA precursor to the fully imidized polyimide structure across all dianhydride compositions was confirmed by FT-IR spectroscopy, as shown in Figure S5. FT-IR spectra were recorded using a Nicolet 5700 spectrometer (Thermo Fisher Scientific, USA) in the range of 600–4000 cm⁻¹. Four characteristic absorption bands associated with the imide ring structure are highlighted in Figure S5.

The asymmetric C=O stretching band of the imide carbonyl appeared at approximately 1780 cm⁻¹, and the symmetric C=O stretching band was observed at approximately 1720 cm⁻¹. These two imide carbonyl bands are the most diagnostic features of the fully cyclized imide ring, arising from the in-phase and out-of-phase stretching vibrations of the two C=O groups flanking the imide nitrogen. The C–N stretching band characteristic of the imide ring was confirmed at approximately 1380 cm⁻¹, and the imide ring deformation band appeared near 720 cm⁻¹. In addition, a pronounced absorption band observed at approximately 2920 cm⁻¹ was assigned to the aliphatic C–H stretching vibrations contributed by the long hydrocarbon chain of DDA, confirming the incorporation of the aliphatic diamine segment into the polyimide backbone.

Critically, the absence of the broad N–H stretching band (~3300 cm⁻¹) and the amide C=O band (~1660 cm⁻¹) characteristic of the PAA precursor in all four spectra confirms that the thermal imidization cycle was completed effectively regardless of the dianhydride composition. These results confirm that variations in the ISSDA:ODPA ratio under the applied imidization conditions do not inhibit the ring-closure reaction, and that all films examined in the main text represent the fully imidized polyimide structure rather than partially cyclized PAA intermediates.

**Calculation of Theoretical Bio-Based Carbon Content**

The theoretical bio-based carbon content (CB) of the optimized composition (ISSDA:ODPA = 6:4, Cys:DDA = 5:5) was calculated from the known bio- or fossil-derived origin of the carbon atoms in each monomer, as a structural estimate consistent with the concept measured experimentally by ASTM D6866.

ISSDA (24 carbons total) contains a bio-derived isosorbide core (6 carbons) esterified with two petroleum-derived trimellitic anhydride units (9 carbons each); only the isosorbide-derived carbons were counted as bio-based. DDA (Priamine 1075, 36 carbons) is a fully bio-based C36 dimer diamine. ODPA (16 carbons) and Cys (4 carbons) were treated as entirely non-bio-based.

Weighting each monomer by its mole fraction in the optimized composition: Total carbon = 0.6(24) + 0.4(16) + 0.5(4) + 0.5(36) = 40.8; Bio-based carbon = 0.6(6) + 0.5(36) = 21.6. This gives CB ≈ 21.6/40.8 × 100% ≈ 52.9%.

**Figure S5.** (a) Synthetic scheme for the preparation of self-healable polyimide (SHPI) films via poly(amic acid) formation and stepwise thermal imidization. (b) FT-IR spectra of polyimide films with varying ISSDA:ODPA molar ratios at a fixed Cys:DDA ratio of 5:5. Yellow highlighted regions indicate the characteristic imide absorption bands at approximately 1780 and 1720 cm⁻¹ (imide C=O stretching), 1380 cm⁻¹ (imide C–N stretching), 2920 cm⁻¹ (aliphatic C–H stretching from DDA), and 720 cm⁻¹ (imide ring deformation).

**S6. Enlarged Photographic Comparison of Optical Transparency**

Figure S6 presents enlarged photographs of two representative polyimide films placed over a printed background image to provide a direct and visually unambiguous comparison of their optical transparency. Both films were prepared at a fixed Cys:DDA ratio of 5:5, with panel (a) showing the ISSDA-rich composition (ISSDA:ODPA = 6:4) and panel (b) showing the ODPA-only composition (ISSDA:ODPA = 0:10). These photographs correspond to the same two films shown in Figure 2(b) of the main text and were taken under identical lighting conditions to more clearly illustrate the transparency difference that is also captured in the UV–vis transmittance spectra of Figure 2(a).

As clearly shown in Figure S6, the ODPA-only film (panel b) renders the background noticeably blurred and imparts a distinct yellowish tint, consistent with the lower transmittance and red-shifted absorption onset observed in the UV–vis spectrum of this composition. The yellowish coloration is a well-known manifestation of charge-transfer complex (CTC) formation between the electron-donating diamine units and the electron-accepting dianhydride segments in conventional aromatic polyimide systems.^S4-S5^ In stark contrast, the ISSDA-containing film (panel a) allows the background text and graphics to be distinctly resolved through the film with high optical clarity, demonstrating that the incorporation of the non-planar, sterically bulky ISSDA unit effectively suppresses CTC formation and interchain electronic interactions.

The visual comparison in Figure S6 thus provides direct photographic evidence that the incorporation of ISSDA does not merely produce a marginal improvement in spectroscopic transmittance values, but results in a perceptually and practically significant enhancement in optical clarity. This observation reinforces the interpretation presented in the main text that ISSDA functions as a key structural element for achieving high transparency in the present self-healable polyimide system, rather than serving merely as a minor compositional modifier.

**Figure S6.** Enlarged photographs of polyimide films with (a) ISSDA:ODPA = 6:4 and (b) ISSDA:ODPA = 0:10 at a fixed Cys:DDA ratio of 5:5, placed over a printed background to visualize the difference in optical transparency. The ISSDA-containing film exhibits markedly higher clarity and lower coloration compared with the ODPA-only counterpart. (scale bars: 1.5 cm)

**S7. Healing Efficiency Calculat**

The healing efficiency (HE) of the present polyimide films was quantitatively evaluated by monitoring the change in scratch width during t**ion**he thermal healing process using optical microscopy. Scratch width measurements were performed at defined time intervals during healing, and the HE was calculated according to the following equation:

$$\text{HE}=\left[ 1 - \frac{W_{t}}{W_{0}} \right]\times100\%$$

where *W*₀ represents the initial scratch width immediately after damage and *W*t represents the scratch width measured after a given healing time *t*. A HE value of 100% therefore corresponds to complete crack closure, in which the scratch width is reduced to zero and the damaged surface is fully recovered at the microscopic level. This scratch-width-based evaluation method provides a direct and visually quantifiable measure of the crack closure kinetics, and is consistent with previously reported approaches for assessing optical microscopy-based healing efficiency in self-healing polymer films.

It should be noted that this metric quantifies the geometric closure of the crack rather than the recovery of bulk mechanical properties. Accordingly, the healing efficiency values reported in Figure 4(c) and (d) of the main text reflect the degree of morphological recovery at the damaged interface, and should be interpreted in conjunction with the tensile property data presented in Section 2.6, which provide a complementary assessment of the mechanical recovery after healing.

**S8. SEM Characterization of Silver Nanowires**

Figure S7 presents a scanning electron microscopy (SEM) image of the silver nanowires (AgNWs) used in the electrical self-healing demonstration described in Section 2.7. The AgNWs exhibit a well-defined one-dimensional wire morphology with an average diameter of approximately 300 nm and an average length of approximately 15 μm, yielding a high aspect ratio that is favorable for the formation of a percolating conductive network at low nanowire loading. The uniform diameter and smooth wire surfaces observed in the SEM image confirm the structural integrity of the AgNWs prior to deposition onto the polyimide substrate.

**Figure S7.** SEM image of the silver nanowires (AgNWs) used for constructing the conductive network on the self-healable polyimide film. The AgNWs exhibit an average diameter of approximately 300 nm and an average length of approximately 15 μm (scale bar: 2 μm).

**S9. Computational Details for DFT/TD-DFT Analysis**

Additional computational analysis was performed to support the DFT/TD-DFT results discussed in Figure 3 of the main text. Symmetric, single-chain ODPA- and ISSDA-based polyimide model structures were constructed to compare the effects of dianhydride identity on electronic-transition characteristics, independent of interchain packing. Both the ODPA-based model (ODPA-Cys-ODPA-DDA) and the ISSDA-based model (ISSDA-Cys-ISSDA-DDA) contain two dianhydride units, one Cys unit, and one DDA unit in an identical sequence, differing only in the dianhydride component; this design ensures that any difference in the calculated electronic structure reflects the dianhydride identity rather than a difference in chain length. Because Priamine 1075 (DDA) is a technical-grade mixture of branched C36 isomers without a single well-defined structure, its aliphatic segment was represented in both models by a simplified branched fragment rather than the exact isomer distribution; this simplification is stated here as an explicit assumption.

Geometry optimizations were carried out at the B3LYP/6-311+G(2d,p) level without symmetry constraints using Gaussian 16, followed by frequency calculations confirming the absence of imaginary frequencies for both optimized structures. Because both models are single, continuous covalent chains rather than non-covalently associated pairs, no interchain distance was calculated or is reported. TD-DFT calculations were performed at the CAM-B3LYP/6-311G(d,p) level over the fifty lowest singlet excited states. Semi-local functionals such as B3LYP are known to be unreliable for describing long-range, through-space charge-transfer states between weakly coupled fragments, owing to self-interaction error and the incorrect long-range behavior of the exchange potential;^S6^ range-separated functionals such as CAM-B3LYP substantially correct this deficiency and were therefore adopted for the TD-DFT calculations reported here, following an independent re-examination that identified this limitation in our originally reported B3LYP/6-31G(d,p) results.

Frontier molecular orbital distributions, HOMO and LUMO energy levels, and HOMO–LUMO energy gaps were obtained from the optimized single-chain structures. The ODPA-based model exhibited HOMO and LUMO energy levels of −8.27 and −1.46 eV, respectively (HOMO–LUMO gap = 6.81 eV), with both orbitals co-localized near the Cys disulfide linkage and its adjacent imide carbonyl, consistent with a local, through-bond transition. The ISSDA-based model exhibited HOMO and LUMO energy levels of −8.33 and −1.87 eV, respectively (HOMO–LUMO gap = 6.46 eV), comparable in magnitude to the ODPA-based model; the HOMO and LUMO of the ISSDA-based model were found to be spatially adjacent, in contrast to our previously reported B3LYP/6-31G(d,p) result, in which the frontier orbitals of the ISSDA-based model appeared separated across the two ISSDA termini. We attribute this discrepancy to the self-interaction error inherent to semi-local functionals noted above, which is substantially corrected by the range-separated CAM-B3LYP functional used in the present analysis.

The TD-DFT-calculated absorption spectrum of the ODPA-based model (Figure 3b) shows a dominant transition at 209.05 nm (f = 0.856) together with a weaker, secondary transition at 286.65 nm (f = 0.149). The ISSDA-based model (Figure 3e) shows an intense transition at 206.01 nm (f = 1.925), substantially stronger than the corresponding transition in the ODPA-based model, together with a weaker transition at 261.93 nm (f = 0.082). The presence of this very strong transition in the ISSDA-based model was verified by repeating the TD-DFT calculation with a different number of requested states (45 instead of 50); the transition reproduced at the same wavelength and oscillator strength to four significant figures, confirming that it is not a numerical artifact arising from near-degenerate state mixing.

Because this transition (206 nm) lies substantially outside the near-UV/visible range relevant to film transparency, its potential contribution to near-UV absorption was evaluated quantitatively. Approximating each calculated transition as a Gaussian band with a full width at half maximum (FWHM) of 8 nm (consistent with the linewidth used to generate the convolved spectra in Figure 3b,e), the contribution of the 206 nm transition at wavelengths ≥ 250 nm is more than thirteen linewidths from the band center and is therefore negligible (relative intensity < 10⁻¹⁵ compared with the peak maximum). This transition and its ODPA counterpart at 209 nm are accordingly excluded from the near-UV transparency comparison discussed in the main text.

Restricting the comparison to transitions at λ ≥ 250 nm, the ODPA-based model shows a maximum oscillator strength of 0.149 (286.65 nm; Σf = 0.344 over 19 states in this range), whereas the ISSDA-based model shows a maximum oscillator strength of 0.082 (261.93 nm; Σf = 0.291 over 19 states in this range) at a shorter wavelength. This indicates that, within the near-UV region relevant to transparency, the ISSDA-based model shows both a lower transition intensity and a blue-shifted dominant transition relative to the ODPA-based model. Taken together, these results indicate that the enhanced transparency of the ISSDA-containing polyimides in the near-UV/visible region arises from a combination of reduced transition intensity and a blue shift of the dominant near-UV transition. The interchain packing argument discussed in the main text is supported by the experimental WAXD data (Figure 3g,h) rather than by any dimer-based computational model.

**Reference**

**[S1]** Sawada, R. and S. Ando, Colorless, Low Dielectric, and Optically Active Semialicyclic Polyimides Incorporating a Biobased Isosorbide Moiety in the Main Chain. Macromolecules, 2022. 55(15): p. 6787–6800.

**[S2]** Sawada, R. and S. Ando, Enhancing optical, dielectric, and thermal properties of bio-based polyimides incorporating isomannide with a bent and sterically constrained conformation. Journal of Materials Chemistry C, 2023. 11(43): p. 15053–15064.

**[S3]** Li, X., Y. Zhai, K. Yang, J. Bai, Y. Qiu, and Y. Wang, Preparation and Characterization of a Novel Self-Healing Transparent Polyimide Film Based on Dynamic Disulfide Bonds. Polymers, 2024. 16: p. 3461.

**[S4]** Kim, Y.N., J. Lee, Y.-O. Kim, J. Kim, H. Han, and Y.C. Jung, Colorless polyimides with excellent optical transparency and self-healing properties based on multi-exchange dynamic network. Applied Materials Today, 2021. 25: p. 101226.

**[S5]** Choi, M.-C., J. Wakita, C.-S. Ha, and S. Ando, Highly Transparent and Refractive Polyimides with Controlled Molecular Structure by Chlorine Side Groups. Macromolecules, 2009. 42(14): p. 5112–5120.

**[S6]** Dreuw, A. and M. Head-Gordon, Failure of Time-Dependent Density Functional Theory for Long-Range Charge-Transfer Excited States. Journal of the American Chemical Society, 2004. 126(12): p. 4007–4016.
